# Mapping of a novel clubroot disease resistance locus in *Brassica napus* and related functional identification

**DOI:** 10.3389/fpls.2022.1014376

**Published:** 2022-09-28

**Authors:** Xuefei Jiang, Ying Su, Maolin Wang

**Affiliations:** Key Laboratory of Bio-Resource and Eco-Environment of Ministry of Education, College of Life Sciences, Sichuan University, Chengdu, China

**Keywords:** clubroot disease, mapping, *Plasmodiophora brassicae*, *Brassica napus*, ERF transcription factor

## Abstract

Clubroot disease, caused by *Plasmodiophora brassicae*, is a devastating disease that results in substantial yield loss in *Brassicaceae* crops worldwide. In this study, we identified a clubroot disease resistance (CR) *Brassica napus*, “Kc84R,” which was obtained by mutation breeding. Genetic analysis revealed that the CR trait of “Kc84R” was controlled by a single dominant locus. We used the bulked segregant analysis sequencing (BSA-seq) approach, combined with genetic mapping based on single nucleotide polymorphism (SNP) markers to identify CR loci from the F_2_ population derived from crossing CR “Kc84R” and clubroot susceptible “855S.” The CR locus was mapped to a region between markers *Bn*SNP14198336 and *Bn*SNP14462201 on the A03 chromosome, and this fragment of 267 kb contained 68 annotated candidate genes. Furthermore, we performed the CR relation screening of candidate genes with the model species Arabidopsis. An ERF family transcriptional activator, *Bn*ERF034, was identified to be associated with the CR, and the corresponding Arabidopsis homozygous knockout mutants exhibited more pronounced resistance compared with the wild-type Col-0 and the transgenic lines of *BnERF034* in response to *P. brassicae* infection. Additionally, the expression analysis between resistant and susceptible materials indicated that *BnERF034* was identified to be the most likely CR candidate for the resistance in Kc84R. To conclude, this study reveals a novel gene responsible for CR. Further analysis of *BnERF034* may reveal the molecular mechanisms underlying the CR of plants and provide a theoretical basis for *Brassicaceae* resistance breeding.

## 1 Introduction

Clubroot disease caused by *Plasmodiophora brassicae* is one of the most devastating diseases affecting *Brassicaceae* crops, it is widespread throughout the world and causes significant economic losses every year (Dixon, 2009). *Brassica napus*, the largest planted oil crop in the *Brassicaceae* family, has been particularly badly affected (Hejna et al., 2019). *P. brassicae* is difficult to eradicate once the fields are contaminated because it can remain in the soil for years as resting spores. Furthermore, there are currently no good control measures for agricultural production (Webster and Dixon, 1991; Wallenhammar, 2010). On the whole, resistance breeding is one of the most effective and desirable approaches to deal with this disease (Piao et al., 2009).

Although there are many crops in the *Brassicaceae* family, limited resources are identified for clubroot disease resistance (CR) (Jiang et al., 2018). CR genes in *Brassica rapa* have been identified most frequently and studied most intensively (Sakamoto et al., 2008), among which two most well-studied CR genes, *CRa* (A03) (Matsumoto et al., 1998; Hayashida et al., 2008) and *Crr1a* (Hatakeyama et al., 2013) (A08), have been successfully isolated (Ueno et al., 2012). In addition, there are many CR loci for genes of undefined function, such as *Crr2* (Suwabe et al., 2003), *PbBa1.1* (Chen et al., 2013), *QS_B1.1* (Pang et al., 2014) (A01); *CRc* (Sakamoto et al., 2008), *Rcr8* (Yu et al., 2017) (A02); *PbBa3.1* (Chen et al., 2013), *PbBa3.3* (Chen et al., 2013), *Crr3* (Saito et al., 2006), *CRd* (Pang et al., 2018), *CRk* (Sakamoto et al., 2008), *CRb* (Kato et al., 2013), *QS_B3.1* (Pang et al., 2014), *Rcr1* (Yu et al., 2017), *Rcr4* (Yu et al., 2017), *Rpb1* (Chu et al., 2013) (A03); *Crr4* (Suwabe et al., 2006) (A06); *CRs* (Laila et al., 2019), *Crr1b, PbBa8.1* (Chen et al., 2013), *Rcr9* (Yu et al., 2017) (A08), and most of these loci contain typical disease resistance proteins. In *Brassica oleracea*, relatively few CR loci have been reported (Piao et al., 2009; Ce et al., 2021), such as *CR2a* (Grandclément and Thomas, 1996), *CR2b* (Grandclément and Thomas, 1996), *Pb3* (Voorrips et al., 1997), *Pb4* (Voorrips et al., 1997) and *PbBo1* (Rocherieux et al., 2004), and most of them exhibit quantitative traits controlled by polygenic. In *B. napus*, it appears to have many disease resistance loci (more than 30 CR loci) reported (Hejna et al., 2019), but these are either less (only two) dominant loci or from a single source (most of CR loci have been mapped by Werner et al.) (Werner et al., 2008). Notably that all these CR loci are still poorly studied in terms of disease resistance function and resistance mechanisms.

At present, the utilization of CR loci primarily uses intraspecific or interspecific mating to generate new CR materials. For instance, Sakamoto and Suwabe et al. have developed numerous CR cabbages using European fodder turnips as a source of resistance (Suwabe et al., 2003; Sakamoto et al., 2008). Because *B. napus* is a natural amphidiploid between *B. oleracea* and *B. rapa* (Piao et al., 2009), Diederichsen et al. used the interspecific cross of CR turnip and CR kale material to produce two novel CR *B. napus*, “Mendel” and “Tosca” (Diederichsen and Sacristan, 1996; Diederichsen et al., 2006), which were found to be resistant to the clubroot disease both in the laboratory and field identification. Furthermore, Zhan et al. developed a new conventional variety, Huashuang 5R, and a new hybrid variety, Huayouza 62R, with excellent CR by interspecific crosses of *B. napus* material containing the resistance locus *PbBa8.1* with CR turnips ECD04 and CR Chinese cabbage, respectively (Zhan et al., 2020, Li et al., 2021b). Shah et al. combined two CR genes, *CRb* and *PbBa8.1*, which originated from two homozygous *B. napus* CR varieties 305R and 409R, respectively, and developed CR homozygous lines through marker-assisted selection (MAS) (Shah et al., 2019). However, in general, CR resources, especially in *B. napus*, are still scarce and the use of resistance loci is mostly limited by the source of resistance. And the interspecific crosses require a long breeding cycle, so it is still important to identify CR resources from *B. napus* for the creating of varieties of *B. napus* resistant to clubroot disease.

In our study, a novel CR material, “Kc84R,” and a susceptible material, “855S,” of *B. napus* were used to develop a new segregation population. We performed the inheritance analysis to determine that the CR trait of Kc84R is a qualitative trait controlled by a dominant locus. The CR loci were identified by bulk segregation analysis sequencing (BSA-Seq) strategy, which is based on genome resequencing in two sets of extreme pools. The target region was then gradually narrowed and located on a 267 kb fragment of the A03 chromosome. Furthermore, we performed the CR relation screening of candidate genes with the model species Arabidopsis. A candidate gene, *BnERF034*, encoding a transcriptional activator, was shown to be strongly associated with disease resistance. Our study identifies a new type of CR gene, and provides important information for future resistance breeding in *Brassicaceae* species.

## 2 Materials and methods

### 2.1 Plant materials and pathogen isolates

Two *B. napus* materials: CR material (previously, a *B*. *napus* material was screened out with excellent resistance to a *P. brassicae* isolate of Sichuan Guanghan during years of field cultivation) “Kc84R,” a selected line of Kc84-1, which comes from the “2013-84” mutagenized by Atmospheric and Room Temperature Plasma (ARTP), followed by more than six generations of successive self-pollinated and selection pressure of clubroot disease in field cultivation; susceptible material “855S,” which verified by field cultivation, were selected as parents to produce F_1_ progenies, and F_1_ plants were self-pollinated to develop F_2_ segregating population.

*Arabidopsis thaliana*: Wild-type Columbia-0 (Col-0), the mutant T-DNA single insertion lines *wrky12* (salk_204550c), *erf034* (salk_087049c), *atg8e* (salk_126394c), *tcp9* (salk_201398c), *urm1* (cs812600), *pub15* (salk_120989c), *skp1* (salk_042202) in Col-0 background were obtained from Arabidopsis Biological Resource Center (http://www.arabidopsis.org/abrc/), *pme17* (salk_059908c) was obtained from Arashare (https://www.arashare.cn/index/). Homozygous mutant plants were selected and tested for T-DNA insertion and homozygosity by PCR using primers obtained by T-DNA Primer Design Tool (http://signal.salk.edu/tdnaprimers.2.html) (**Supplementary Table 1**).

The *P. brassicae* pathotypes: *P. brassicae* infection tests were comprised of pathotypes 2, 4, 7, and 11 in Williams system supplied by Piao, in addition, the local *P. brassicae* isolate collected from Guanghan, Sichuan Province, China (E104.1797, N31.0382), named it *Pb*Gh, which has been identified as Pb3, according to the sinitic clubroot differential set (SCD) system (Pang et al., 2020).

### 2.2 Pathogen preparation and inoculation

All pathogen isolates were propagated with kinds of *B. napus* materials, and fresh galls were stored at −20°C for resting spore extraction. Preparation of *P. brassicae* resting spores was taken with sucrose gradient centrifugation. In brief, the infected plant galls were homogenized with sterile water using a grinder after surface cleaning and sterilization. The homogenate was filtered through 8 layers of cheesecloth, then the filtrate was collected and centrifuged at 3 100 rpm, the sediment was washed several times with sterile water and suspended in 50% sucrose solution. The supernatant was retained after centrifugation, and then centrifuged with additional sterile water. The sediment was collected, and washed several times again, the resting spores were obtained.

In soil culture infection, the inoculation of resting spores was taken with the injection. Firstly, holes were dug in the substrate to be transplanted, and 2 g sterilized vermiculite was added. Then, 1 mL of adjusted concentrations of *P. brassicae* resting spores were carefully injected into the vermiculite, and the roots of plants to be transplanted were then wrapped in the infiltrated vermiculite. The inoculation concentration depended on different experiments.

In hydroponic culture infection, ½ Hogland nutrient solution was used as the medium with the same temperature and light conditions. The resting spores of *P. brassicae* were diluted into 1×10^7^ mL^-1^. *A. thaliana* was treated in 6 well cell culture plates, and *B. napus* was placed in 50 mL conical flasks.

### 2.3 Disease index evaluation and phenotypic investigation

Disease symptoms of *B. napus* were evaluated at 28 days post inoculation (dpi) with *P. brassicae*. After thorough cleaning of the roots, disease symptoms were scored as follows: 0, no obvious galls; 1, a few small galls mainly on lateral roots; 3, larger galls on the lateral roots or small galls on the main roots; and 5, large galls both on the lateral and main roots (Huang et al., 2017) (**Supplementary Figure 1**).

Symptoms of *A. thaliana* were assessed on a 0–5 scale at 21 and 28 dpi, respectively, where 0, no obvious galls; 1, small galls mainly on lateral roots; 3, obvious galls on the main roots and plant growth were inhibited; 5, severe galls on the whole roots and extending to the hypocotyl, plant growth was severely affected (**Supplementary Figure 2**). The disease index (DI) was calculated as following:


$$\text{DI }\left(\%\right)=\frac{\sum^{}\left(\text{rating class}\right)\times \;\left(\#\text{ plants in rating class}\right)\;}{\left(\text{total }\#\text{ plants in treatment}\right)\times \;5}\;\times \;100$$

Hypocotyl width: the plants used to calculate hypocotyl width were selected randomly from trays, with 4 plants per tray for each material.

Hydroponic culture infection observation: the infection phenotype of the root hairs and main roots of *A. thaliana* that need to be observed were obtained from corresponding sampling points. The phenotypes were photographed with microscope (Nikon DS-Ri2, Tokyo, Japan).

### 2.4 Selection of pathogen pathotypes for mapping

“Kc84R” was inoculated with *P. brassicae* pathotypes 2, 4, 7, 11 and the local *P. brassicae* isolate, respectively. “855S” was inoculated with the *P. brassicae* pathotypes that “Kc84R” showed disease resistance in previous tests. The concentration of resting spores was 1 × 10^7^/mL.

### 2.5 Population genetic analysis

“Kc84R,” “855S,” the F_1_ population from 855S (female) × “Kc84R” (male) (more than 60 plants), and the segregating population obtained from F_1_ self-fertilization (F_2_ lines) (560 plants), were inoculated with *P. brassicae* pathotypes for mapping. The concentration of resting spores was 1 × 10^7^/mL.

### 2.6 BSA-seq analysis

To construct reliable extreme separation pools, the F_2_ segregating populations were randomly divided into 5 groups, in which each group contained about 200 F_2_ lines. Total 20/20 extreme R/S plants were selected from the 5 groups. Individual plants genomic DNA was extracted from young leaves of two parents (10 plants per material) and the extreme lines following the manufacturer’s instructions of Plant Genomic DNA Kit (TIANGEN, Beijing, China). R and S pools were constructed by mixing an equal volume of DNA from the extreme R and S lines within each group, the parental pools were constructed in the same way. The two separation pools and two parental pools were subjected to perform whole-genome sequencing on the illumina HiSeqTM PE150 platform by Novogene (Beijing, China). Read pairs with adapters were removed; the paired reads with N ratio over 10% were removed; the paired reads that low quality (Q ≤ 5) bases exceeds 50% of the length, need to be removed. Then, the clean reads were mapped to the reference genome of *B. napus* (https://ftp.ncbi.nlm.nih.gov/genomes/genbank/plant/Brassica_napus/latest_assembly_versions/GCA_000686985.2_Bra_napus_v2.0/) with BWA (mem -t 4 -k 32 -M), followed by de-duplication with SAMTOOLS (rmdup).

### 2.7 SNP discovery and mapping

Clean reads were used for the detection of SNPs and InDels. The detection of multiple sample SNPs with the Unified Genotyper module of GATK3.8 software, Variant Filtration for filtering with the parameters –cluster Window Size 4, –filter Expression “QD< 4.0 || FS > 60.0 || MQ< 40.0,” -G_filter “GQ<20.” The InDels detection also used Unified Genotyper module of GATK3.8 software, Variant Filtration for filtering with the filter parameter –filter Expression “QD< 4.0 || FS > 200.0.” SNP and InDel annotation using ANNOVAR. The parents were selected as a reference, and SNP/InDel-index (frequency of SNPs/InDels) was calculated for each SNP/InDel loci between the parents and R/S pools. SNP loci with SNP-index less than 0.3 and SNP depth less than 7 in both R and S were filtered out; SNP loci with a missing SNP-index in one offspring pool were filtered out; InDels loci that were missing in the offspring pools were filtered out.

Δ(SNP/InDel-index) curve: 1 Mb was chosen as the window, 1 kb was chosen as the step size, and the mean of SNP/InDel-index was calculated to reflect the SNP/InDel-index distribution of the two offspring pools in each window. Δ(SNP/InDel-index) = SNP/InDel index (extreme trait B) - SNP/InDel index (extreme trait A), 1,000 permutation tests were performed and the 95% confidence level was selected as the threshold for screening. The preliminary candidate interval was confirmed by the Δ(SNP/InDel-index) curve.

### 2.8 SNP genotyping and identification of target region

Appropriate SNP/InDel loci (17 SNPs and 3 InDels) were selected as markers on target chromosomes (**Supplementary Table 2**), especially candidate intervals, based on SNP/InDel_index in BSA-seq. SNP genotyping of the 810 F_2_ lines was performed by SNaPshot and genetic distances were calculated using QTL IciMapping (Version 4.2.53) with the F_2_ population approach for genetic distance calculation, with parameters set to default. The primers used are listed in **Supplementary Table 3**.

### 2.9 Gene synteny analysis of the target region

*B. napus* ZS11 genome (Bra_napus_v2.0) and *A. thaliana* genome (Arabidopsis_thaliana. TAIR 10) were downloaded from the NCBI and ENA (European Nucleotide Archive) respectively. Then, the sequences and GFFs of each genome were extracted by One-Step MCScanX module of TBtools (Version 1.09876) with parameter set as default. Finally, the gene synteny analysis was performed with the CDS of the candidate chromosome as queries and CDS of the *A. thaliana* genome as subject with Dual Systeny Plot for MCscan X (Chen et al., 2020).

### 2.10 Quantitative RT-PCR

The whole roots were collected from “Kc84R” and “855S” at 14 and 28 dpi with *P. brassicae* in soil, and with three biological replications for RNA extraction, each repeat contains 3-6 roots. The whole roots were collected from “Kc84R” and “855S” at 12 and 24 hours, 3-, 5-, and 7-days post inoculation with *P. brassicae* in hydroponic culture, and with three biological replications for RNA extraction, each repeat contains 6-9 roots. RNAprep Pure Kit TSP411 (TSINGKE; Beijing, China) with on-column deoxyribonuclease (DNase) digestion was used for RNA extraction from each sample following the manufacturer’s instructions. The RNA concentration and quality were checked using a Nano-400A Micro-Spectrophotometer (ALLSHENG, Hangzhou, China). The preparation of cDNA libraries performed by *TransScript* ^®^ Uni All-in-One First-Strand cDNA Synthesis SuperMix for qPCR (One-Step gDNA Removal) (Transgene; Beijing, China). PCR was conducted using the Taq Pro Universal SYBR qPCR Master Mix (Vazyme; Nanjing, China) following manufacturer’s instruction. Cycling conditions were 95°C for initial 30 s followed by 42 cycles of 10 s at 95°C, 30 s at 60°C. Melt-curve profiling and agarose gel electrophoresis was conducted to evaluate the specificity of the reaction and the absence of primer dimers. *BnACTIN* was used as an endogenous control to normalize the expression levels of target genes. The primers used in this part are listed in **Supplementary Table 4**.

### 2.11 Bioinformatic analysis of candidate genes

The expression data of *A. thaliana* in response to *P. brassicae* infection was downloaded from the ENA (https://www.ebi.ac.uk/ena/) under the identifier PRJEB12261 (Malinowski et al., 2016; Olszak et al., 2019).

The visual expression data of the target genes in *A. thaliana* and *B. napus* were downloaded from eFP Browser (http://bar.utoronto.ca/eplant/) and *Bn*TAIR (*Brassica napus* transcriptome information resource) (http://yanglab.hzau.edu.cn/BnTIR/eFP).

The protein spatial structure of the target gene is downloaded from Swiss-model (https://swissmodel.expasy.org/), and made by PyMol (https://pymol.org/2/).

### 2.12 Subcellular localization

The full-length coding sequences of *Bn*ERF034-855S and *Bn*ERF034-Kc84R were fused with eGFP at C-terminus and inserted into the pBI121 vector, respectively. The primers used are included in **Supplementary Table 4**. Then, the recombinant vectors and pBI121 were transformed into *A. tumefaciens* strain GV3101, with infiltration into 4- to 6-week-old *Nicotiana benthamiana* leaves. *N. benthamiana* leaves were harvested at 2 days post infiltration (dpi) and imaged using a fluorescence microscope (Leica DM4 B, Weztlar, Germany).

### 2.13 Transactivation assay in yeast cells

The yeast strain AH109, containing the *lacZ* and *HIS3* reporter genes, was used as an assay system. The yeast vector pGBKT7-BD with DNA binding domain (DNA-BD) but lacking an activation domain was used to verify the transcriptional activation effect of the transcription factor. The coding sequences of *Bn*ERF034-855S and *Bn*ERF034-Kc84R proteins were obtained by PCR. The primers used are included in the **Supplementary Table 4**. Then, the PCR products were cloned into pGBKT7-BD vector containing the GAL4 DNA-binding domain to obtain plasmids for testing of transcriptional activation. The recombinant vectors named “*Bn*ERF034-855S” and “*Bn*ERF034-Kc84R,” the positive control pGAL4 named “GAL4,” the negative control named “PUB15” and the blank group pBD vector, were all transformed into the yeast host strain AH109. All transformants were successively dropped onto YPAD, SD/-Trp and SD/-Trp/-His plates. Transcriptional activation activities were evaluated according to their growth status. Transfected yeast cells were also transferred onto SD/-Trp/-His and incubated in the presence of X-α-gal to check the β-galactosidase activity by monitoring the generation of blue.

### 2.14 Generation of transgenic Arabidopsis plants

The *BnERF034* full-length CDS was amplified from Kc84R and 855S cDNA libraries. PCR products with the restriction sites at both ends were cloned into the binary vector pFGC5941 to produce the constructs *35S::BnERF034* using the methods of enzyme digestion and enzyme ligation. The primers used are listed in the **Supplementary Table 4**. The recombinant vectors were transformed into *A. tumefaciens* GV3101 by the heat shock method. Wild-type Col-0 was transformed using the Agrobacterium- mediated floral dip method (Clough and Bent, 1998). Transgenic T_1_ was selected by spraying 1/1500 Basta solution onto the leaves. PCR and qPCR identification of the surviving positive lines were performed using specific primers of recombinant vectors, the *AtACTIN2* was used as an internal reference control (**Supplementary Table 4**). Ten positive lines were selected from the T_2_ generation with a basta resistance separation ratio of 3:1 to generate homozygous transgenic plants (T_3_) for further analysis.

## 3 Results

### 3.1 Mapping of resistance loci

**Figure 1:**
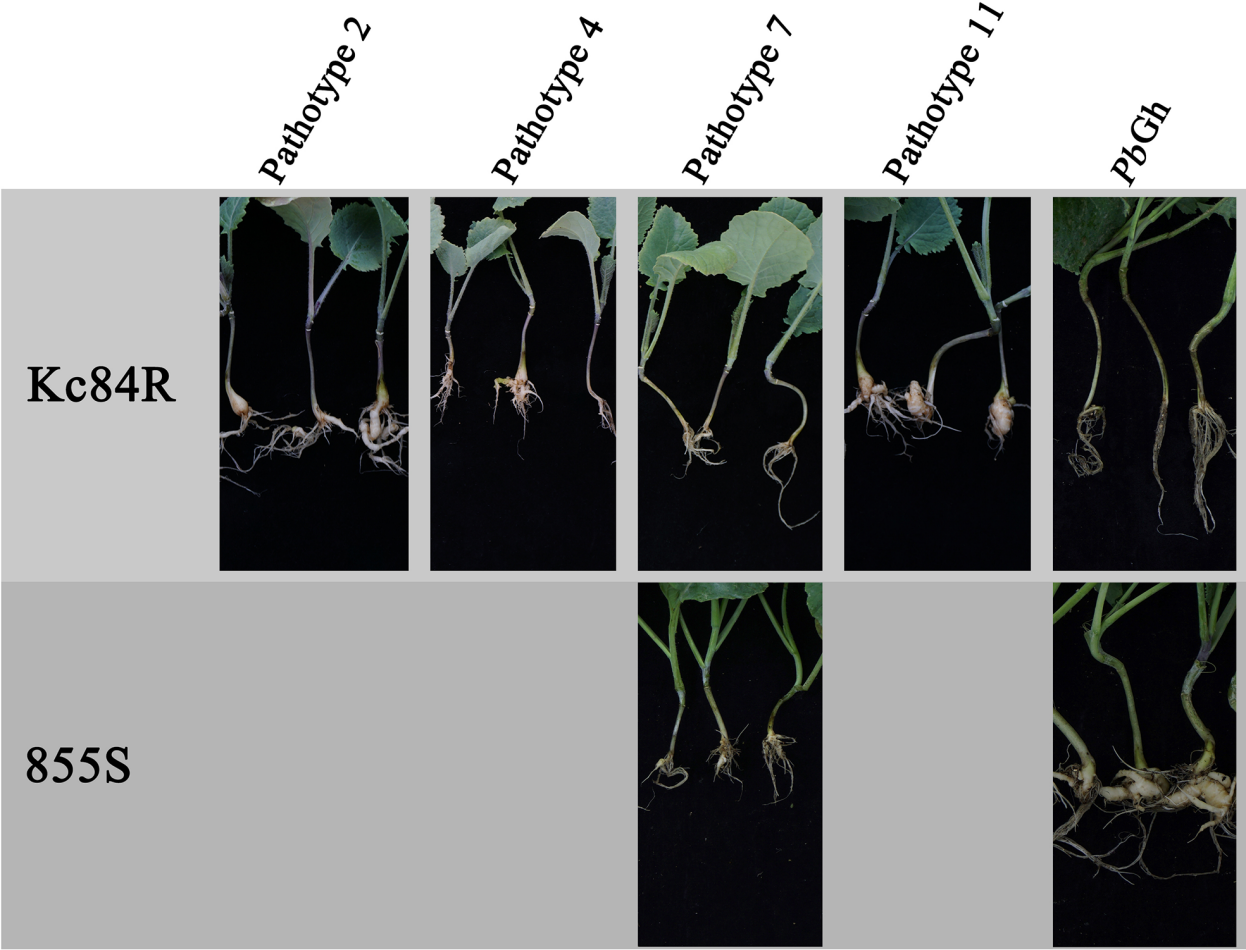
Phenotypes of parent materials to *P. brassicae* pathotypes.

To verify the *P. brassicae* pathotype resistance profile of Kc84R and characterize the identity of CR genes, Kc84R was inoculated respectively by five different *P. brassicae* isolates, which are pathotypes 2, 4, 7, 11 that classified based on the differential system of Williams and *P. brassicae* isolate collected from Guanghan, namely, *Pb*Gh. The disease index was investigated at 28 days post inoculated (dpi). As shown in **Figure 1**, Kc84R showed significant resistance to pathotype 7 (DI 2.83) and *Pb*Gh (DI 1.24), typical susceptibility characteristics to pathotypes 2 (DI 82.31) and 11 (DI 95.76), and lower susceptibility to pathotype 4 (DI 76.33) than pathotypes 2 and 11 (**Table 1**). The susceptible parent material, 855S, used for population construction, was also tested for resistance to several *P. brassicae* isolates (pathotype 7 and *Pb*Gh). The results showed that 855S was significantly susceptible to *Pb*Gh (DI 92), and low susceptibility to the pathotype 7 (DI 18.06) (**Table 1**). Therefore, we selected the *P. brassicae* isolate *Pb*Gh for phenotypic identification of the resistant gene mapping population.

**Table 1:** Disease investigation for susceptibility of parent materials to *P. brassicae* pathotypes.

Materials	Pathotype	DI (Disease Index)
Kc84R	2	82.31 ± 1.74^b^
	4	76.33 ± 4.30^b^
	7	2.83 ± 0.12^d^
	11	95.76 ± 3.26^a^
	*Pb*Gh	1.24 ± 0.81^d^
855S	7	18.06 ± 4.73^c^
	*Pb*Gh	92.00 ± 1.32^a^

Significance analysis of disease index was performed using the one-way ANOVA method, Tukey’s test, and different letters indicate a significant difference (*p* < 0.05).

**Figure 2:**
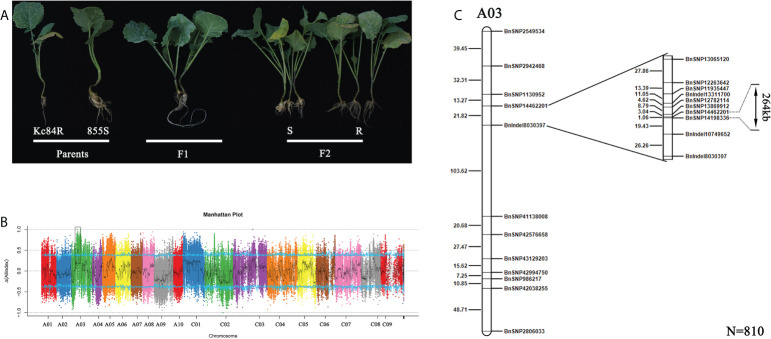
Mapping of candidate CR locus. **(A)** Phenotype identification of two parent lines and their crossed offspring after inoculation with *Pb*Gh. **(B)** The CR loci identified by BSA-sequencing (BSA-seq). **(C)** Fine mapping of the CR locus based on SNP markers using 810 F_2_ individuals. The genetic distance is shown on the left, the physical locations of SNP markers are included in the names.

To confirm the genetic characteristics of the CR loci on Kc84R, we crossed the resistant material Kc84R with the susceptible material 855S and established a segregating population for genetic mapping. And phenotypic identification was performed on the relevant material in the disease-resistant localization population. The results showed that the tested F_1_ generation materials were basically free of disease (**Figure 2A**), while the separation ratio of the F_2_ population roughly conformed to 3:1 (χ^2 ^= 3.125, *P* > 0.05) (**Table 2**). In summary, we inferred that the CR phenotype of Kc84R is a qualitative trait controlled by a dominant locus.

**Table 2:** Disease phenotype statistics in F_1_ population from cross of Kc84R and 855S.

Population	Disease rating				Total	Incidence Rate	χ²	χ²_0.05_
	0	1	3	5				
Kc84R (R parent)	60	1	0	0	61	1.64		
855S (S parent)	0	0	3	61	64	100		
F_1_	64	2	0	0	66	3.03		
F_2_	430	2	12	116	560	23.21	0.0029	3.841

R, resistant; S, susceptible. A rating of “0” was defined as R, “1,” “3,” “5” as S.

To identify the locus that controls the CR trait, BSA-seq based on the second-generation sequencing was carried out. Genomic DNA of two extreme F_2_ pools (R-pool and S-Pool) and two parent pools (Kc84R, 855S) was sequenced. A total of 25,521,831,000 bp and 24,151,904,400 bp raw reads were generated from the R- and S-pools, respectively. The raw reads of 13,317,282,600 bp and 13,233,702,000 bp were obtained from the resistant and susceptible parent pools, respectively. The Q30 are all over 91% (**Supplementary Table 5**). After filtering, more than 98% of the reads passed the quality control standard and remained. The genome of *B. napus* (Bra_napus_v2.0) was mapped, and the overall mapping rates exceeded 97% (**Supplementary Table 6**). SNP/InDel was used to calculate the SNP/InDel frequency distribution (**Supplementary Table 7**), ΔSNPS/InDel was plotted against the genomic positions. According to the ΔSNPS/InDel peak value, an interval of about 2.6 Mb from 11.9 Mb to 14.5 Mb on A03 chromosome was determined, which may be related to the CR gene on Kc84R (**Figure 2B**). To further narrow down the candidate interval, we screened 20 SNP/InDel markers (**Supplementary Table 2**) to genotype 810 F_2_ individual plants. The analysis results showed that the CR locus was located between *Bn*SNP14462201 and *Bn*SNP14198336, in an interval of 1.06 cM (**Figure 2C**).

### 3.2 Identification and expression analysis of candidate genes

*Bn*SNP14198336 was located in the gene LOC106438228, and *Bn*SNP14462201 was located in the gene LOC106438265. The physical distance between these two markers (including these two genes) was 267,362 bases. There were 68 genes annotated in this target region according to reference genome (Bra_napus_v2.0). And in the disease resistant material Kc84R, numerous SNP/InDel events (**Supplementary Table 8**) were included in these candidate genes compared to the reference genome and the susceptible material 855S.

**Figure 3:**
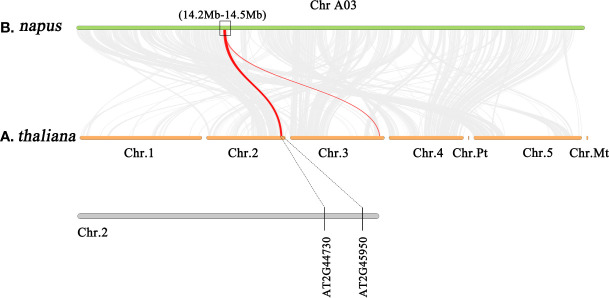
Gene synteny analysis between A03 chromosome of *B*. *napus* and all chromosomes of *A*. *thaliana*.

**Figure 4:**
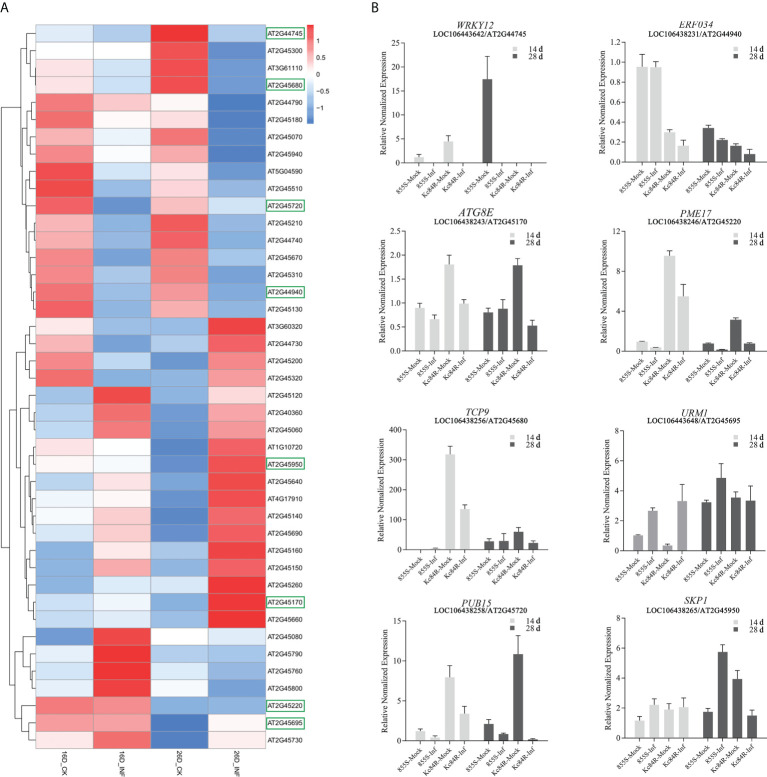
Expression patterns of candidate genes. **(A)** Expression patterns of candidate genes of homologous in *A. thaliana* roots in response to *P. brassicae*, INF indicates the treatment with *P. brassicae*, CK indicates the treatment with sterile water, the boxes indicate genes selected for quantification in *B. napus*; **(B)** transcript levels of selected candidate genes at 14- and 28-days post inoculation (dpi) in *B. napus* Kc84R and 855S. The inoculation with *P. brassicae* was taken as treatment group (Inf) and sterile water as blank control (Mock).

To confirm the sequence characteristics of the candidate genes and perform functional analysis of these candidate genes using *A. thaliana*, we performed covariance analysis of the *B. napus* A03 chromosome with the *A. thaliana* chromosomes. As shown in **Figure 3**, this candidate interval was highly homologous to the interval from AT2G44730 to AT2G45750 on Arabidopsis chromosome 2. The functional annotations of all candidate genes are shown in **Table 3**. Based on the expression data of Arabidopsis in response to *P. brassicae* infection, most genes within the candidate interval exhibited differential expression, some showed high expression and some showed reduced expression levels (**Figure 4A**). Part of differentially expressed genes (LOC106443642, WRKY12-like; LOC106438231, *ERF034*; LOC106438243, *ATG8E*; LOC106438246, *PME17*; LOC106438256, *TCP9*; LOC106443648, *URM1*; LOC106438258, *PUB15*; and LOC106438265, *SKP1*) were selected for further analyses for their expression patterns in response to *P. brassicae* infection in *B. napus* (Kc84R and 855S) (**Figure 4B**). The results showed that the expression level of *WRKY12-like* was low in both Kc84R and 855S. The expression level of *ERF034* in Kc84R was significantly lower than that in 855S, especially in the early stage of infection (14 dpi). The expression level of *ATG8E* in Kc84R was significantly higher than that in 855S in the blank group (Mock), and the down-regulated expression in response to *P. brassicae* infection was also significant. *PME17* is also a gene down-regulated by *P. brassicae* infection, but the overall expression level in Kc84R is significantly higher than that in 855S. At 14 dpi, the expression levels of *TCP9* in Kc84R were much higher than that in 855S. *URM1* is a gene up-regulated at 14 dpi, and the expression level of the two materials was not significantly different. *PUB15* is also a gene down-regulated by *P. brassicae* infection, and the expression level in Kc84R is higher than that in 855S. The differential expression of *SKP1* infected by *P. brassicae* at 14 dpi was not significant, and there was no significant difference between the two materials. These differentially expressed genes, especially those differentially expressed in different materials, may have important relationships with clubroot disease resistance.

**Table 3:** Homologs of candidate genes in *A. thaliana* and functional annotation.

Gene ID	ID in *A. thaliana*	Sort by percent identity (%)	Description of gene functions in *A. thaliana*
LOC106438228	AT2G44730	81.48	Alcohol dehydrogenase transcription factor Myb/SANT-like family protein
LOC106443641	AT2G44740	89.68	cyclin p4;1
LOC106443642	AT2G44745	85.57	WRKY family transcription factor
LOC106441903	AT2G44770	91.61	ELMO/CED-12 family
LOC106438229	AT2G44790	77.99	uclacyanin 2
LOC106438230	AT3G60320	80.50	bZIP domain class transcription factor (DUF630 and DUF632)
LOC106438231	AT2G44940	78.01	Ethylene-responsive transcription factor 34
LOC106441904	AT2G45060	88.52	alanine-tRNA ligase
LOC106438232	AT2G45060	85.02	alanine-tRNA ligase
LOC106438234	AT2G45070	85.88	Preprotein translocase Sec, Sec61-beta subunit protein
LOC106443643	AT2G45080	89.35	cyclin p3;1
LOC106443644	AT2G45110	80.77	expansin B4
LOC106438235	/AT2G40360	85.71	Transducin/WD40 repeat-like superfamily protein
LOC106438238	AT2G45120	79.69	C2H2-like zinc finger protein
LOC106438239	AT2G45130	84.71	SPX domain protein 3
LOC106438240	AT2G45140	87.93	plant VAP homolog 12
LOC106438237	AT2G45150	88.97	cytidinediphosphate diacylglycerol synthase 4
LOC106438236	AT2G45160	79.06	GRAS family transcription factor
LOC106438243	AT2G45170	89.60	AUTOPHAGY 8E
LOC106438242	AT2G45180	86.01	Bifunctional inhibitor/lipid-transfer protein/seed storage 2S albumin superfamily protein
LOC106438241	AT2G45190	88.71	Plant-specific transcription factor YABBY family protein
LOC106438245	AT2G45200	89.00	golgi snare 12
LOC106443645	AT2G45210	77.87	SAUR-like auxin-responsive protein family
LOC106438246	AT2G45220	88.27	Plant invertase/pectin methylesterase inhibitor superfamily
LOC106438247	AT2G45260	85.80	myosin-4 protein (DUF641)
LOC106438248	AT2G45300	89.35	RNA 3’-terminal phosphate cyclase/enolpyruvate transferase, alpha/beta
LOC106438249	AT2G45310	85.23	UDP-D-glucuronate 4-epimerase 4
BNAA03G56780D	AT2G45320	87.82	polyphosphatidylinositol phosphatase
LOC106438251	/AT4G17910	87.25	transferases, transferring acyl groups
LOC106441905	/AT1G10720	24.49	BSD domain-containing protein
LOC106443646	AT2G45510	82.69	cytochrome P450, family 704, subfamily A, polypeptide 2
LOC106438252	AT2G45510	87.38	cytochrome P450, family 704, subfamily A, polypeptide 2
LOC106441906	AT2G45640	85.15	SIN3 associated polypeptide P18
LOC106443647	AT2G45650	86.71	AGAMOUS-like 6
LOC106438253	AT2G45660	93.49	AGAMOUS-like 20
LOC106441907	/		/
LOC106441908	/AT5G04590	86.82	sulfite reductase
LOC111214396	AT2G45670	85.00	lysophosphatidylethanolamine acyltransferase 2
LOC106438255	AT2G45670	87.11	lysophosphatidylethanolamine acyltransferase 2
LOC106438256	AT2G45680	85.80	TCP family transcription factor
LOC106438257	AT2G45690	86.65	shrunken seed protein (SSE1)
LOC106443648	AT2G45695	87.19	Ubiquitin related modifier 1
LOC106443650	AT3G61110	86.92	ribosomal protein S27
LOC106438258	AT2G45720	87.07	ARM repeat superfamily protein
LOC106438259	AT2G45730	88.86	eukaryotic initiation factor 3 gamma subunit family protein
LOC106443651	AT2G45760	77.81	BON association protein 2
LOC106438263	AT2G45790	89.09	Phosphomannomutase (PPM)
LOC106438262	AT2G45800	87.11	GATA type zinc finger transcription factor family protein (PLIM2a)
LOC106438264	AT2G45940	81.40	hypothetical protein (DUF295)
LOC106443652	AT2G45930	75.98	hypothetical protein
LOC106438265	AT2G45950	52.07	SKP1-like 20

“/” represents that the candidate genes in *B. napus* have no homologous sequences in *A. thaliana*.

### 3.3 Resistance function screening of candidate genes

#### 3.3.1 The clubroot disease is significantly reduced in *erf034* mutant during soil culture

Clubroot disease affects most members of the *Brassicaceae* plants, including the model species Arabidopsis, which can conveniently be used to study the biological basis of *P. brassicae*-plant interactions (Walerowski et al., 2018). To further explore the role of these candidate genes in the CR locus, Arabidopsis was taken as the test material. The wild-type *A. thaliana* (Col-0), knockout lines of each candidate gene were obtained and infected by *P. brassicae*. These phenotypes were then recorded and screened. To correctly evaluate the clubroot disease state of *A. thaliana*, we used the Col-0 to establish a rating system for *P. brassicae* infection adapting to our laboratory culture conditions (**Supplementary Figure 2**). According to the phenotypes, two time points, the 21 and 28 dpi were taken as the key disease investigation nodes. The DI, shoot and root phenotype, hypocotyl width and hydroponic infection phenotype were recorded, respectively.

The results of disease investigation showed that the *erf034* mutant (DI 27.5 and 46.25, respectively), corresponding to *ERF034*, had the lowest DI at both 21 and 28 dpi, and was significantly lower than Col-0 plants (DI 49.8 and 76.8, respectively). Other mutants, like *pme17*, *pub15*, and *skp1* also had significantly lower DI than Col-0 plants at 21 dpi. However, the difference between *pme17* and Col-0 was not significant at 28 dpi. While *skp1* and *pub15* had significant differences compared with Col-0, they were still reflected in relatively severe DI. Alternatively, the three mutants *atg8e*, *urm1*, and *tcp9* showed a more severe susceptibility phenotype than Col-0 plants at 21 dpi, but were not significantly different from Col-0 plants at 28 dpi (**Table 4**).

**Table 4:** Results of disease investigation of *A. thaliana* inoculated with *P. brassicae*.

Materials	DI (Disease Index)	
	21 dpi	28 dpi
*Col-0*	49.8 ± 1.35^b^	76.80 ± 2.41^ab^
*atg8e*	60 ± 0.72^a^	79.58 ± 1.05^a^
*erf034*	27.5 ± 0.72^e^	46.25 ± 1.10^d^
*pme17*	43.9 ± 0.74^c^	71.72 ± 1.01^bc^
*pub15*	35.51 ± 1.74^d^	65.97 ± 1.73^c^
*skp1*	37.5 ± 0.36^d^	69.72 ± 0.503^c^
*urm1*	59.22 ± 1.58^a^	80.83 ± 1.27^a^
*tcp9*	63.31 ± 1.17^a^	80.83 ± 0.96^a^

Significance analysis of the disease index in the table was performed using the one-way ANOVA method, and different letters indicate a significant difference (*p* < 0.05).

**Figure 5:**
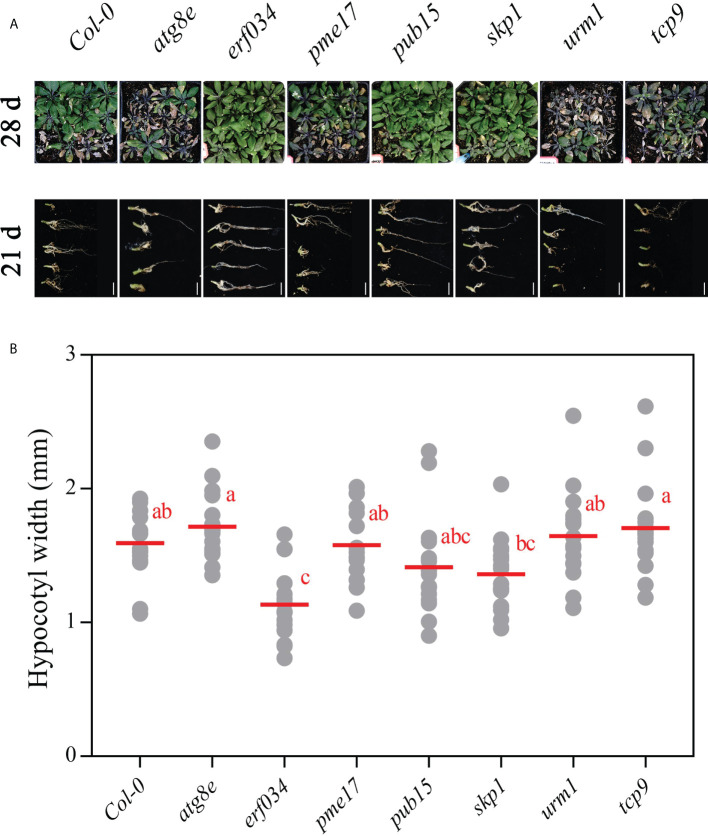
Phenotypes of *A. thaliana* inoculated with *P. brassicae* in soil culture. **(A)** Above-ground parts of Col-0 and each mutant material at 28 dpi, roots at 21 dpi; scale bars represent 2 cm. **(B)** Hypocotyl width of *A. thaliana* inoculated with *P. brassicae* at 21 dpi. Scatter plots present individual hypocotyl width measurements (12 replicates for each treatment), calculated means and SEs. Different letters indicate significant differences between means (Tamhane’s test, *p *< 0.05).

To visualize the phenotypic response of each mutant to *P. brassicae* infection, the roots and above-ground phenotypes of infected plants were photographed at 21 and 28 dpi, respectively (**Figure 5A**). The roots of wild-type Col-0 were significantly expansive growth at 21 dpi, and the ends of some severely infected main roots had started to rot, but most of the relatively healthy lateral roots were still preserved. While the roots of the more severely susceptible *atg8e*, *urm1*, and *tcp9* mutants showed obvious symptoms, such as rot of main roots and disappearance of lateral roots. The roots of *erf034*, *pme17*, *pub15*, and *skp1* mutants were less susceptible, especially the *erf034* mutant, which had robust lateral roots and many roots without obvious infection. The phenotype of the infected plants was more visually reflected in the above-ground parts by 28 dpi. The severely affected plants showed symptoms of wilting and dryness, whereas the less affected plants showed a clear purple coloration and senescence.

At the later stage of *P. brassicae* infection, the root expansion would gradually extend to the hypocotyl, so the thickness of the hypocotyl can also reflect the infection level of host plants. We counted the hypocotyl diameter of each Arabidopsis material at 21 dpi. As shown in **Figure 5B**, the width of *erf034* hypocotyls, expanded by an average 1.08 mm, was the smallest and significantly lower than that of the wild-type Col-0 and other mutants, showing the lowest degree of susceptibility. While the width of the primary roots of some mutants differed did not show significance, which was related to the characteristics of different materials in response to *P. brassicae* infection. Some of the mutants had earlier decay of the primary roots after the onset of infection and could not develop to a later stage, such as *atg8e*, *urm1*.

#### 3.3.2 The development of *P. brassicae* is inhibited in *erf034* mutant during hydroponic culture

**Figure 6:**
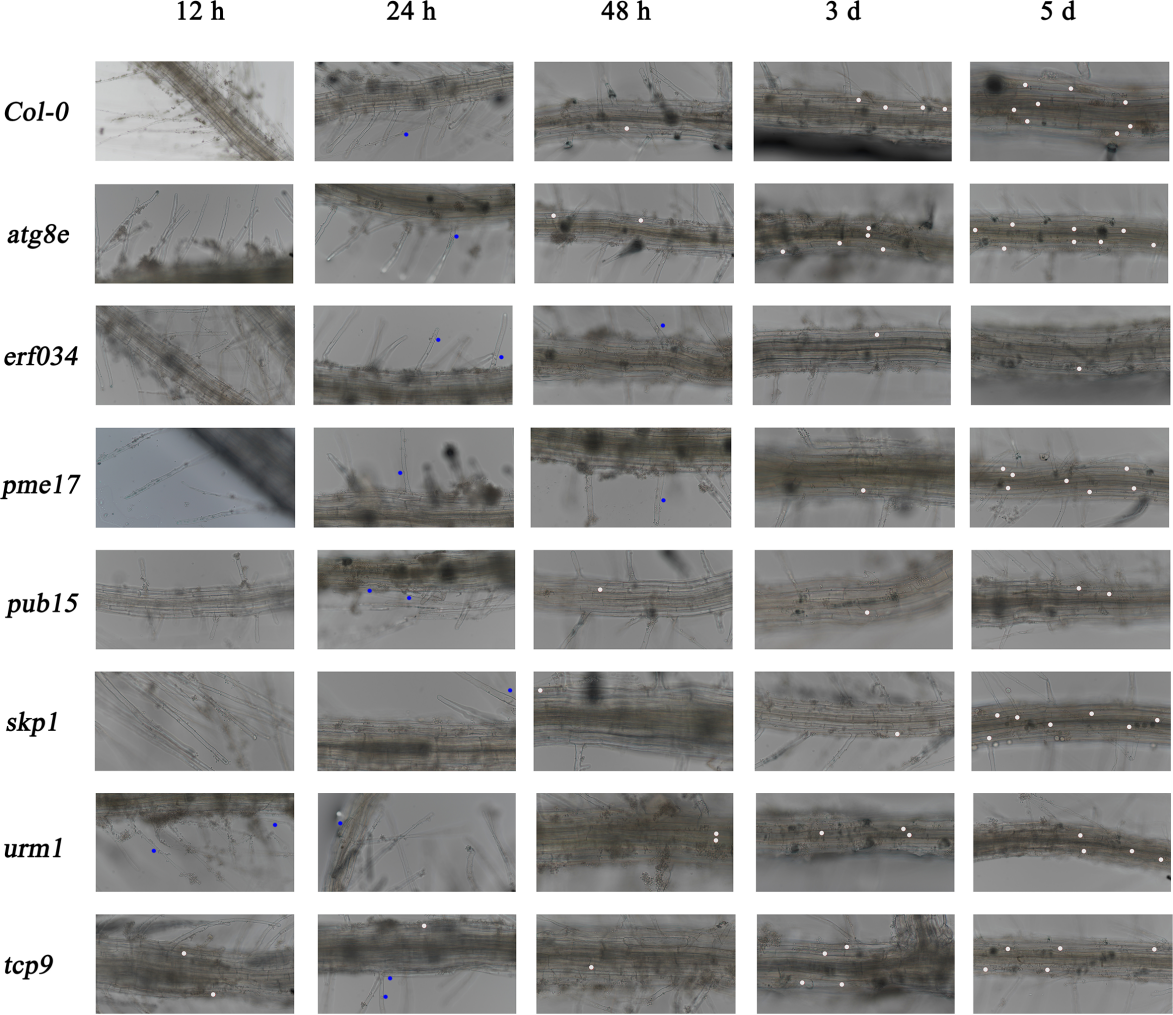
Roots observation of *A. thaliana* inoculated with *P. brassicae* in hydroponic culture. Blue dots indicate the *P. brassicae* of root hair-infected stage, white dots indicate the *P. brassicae* of cortex-infected.

To further determine the differential response of each material in response to *P. brassicae* infection, we conducted hydroponic culture infection experiments on each material. In early infection of *P. brassicae*, root hair infection was the first to occur, but previous studies have shown that root hair infection lacks specificity, and many non-host plants of *P. brassicae* also underwent this link (Ren et al., 2016). For the host plants, successful cortical infection is the key to *P. brassicae* colonization, continuous observation of cortical infection under hydroponic culture can help us determine the differences in resistance levels of different materials to *P. brassicae* infection. As shown in **Figure 6**, wild-type Col-0 was taken as a reference, the occurrence mainly was adsorption of *P. brassicae* resting spores or primary zoospores during the first 12 h of infection. By the 24th hour, root hair infection could already be observed, and some small primary plasmodia began to appear. By the 48th hour, the invasion of *P. brassicae* began to be detected in the cortex cells of Arabidopsis main root. Secondary plasmodium could be observed, and from then on to the period of 3 and 5 dpi, cortical infection gradually increased, and various developmental secondary plasmodia could be observed in cortex cells. Compared with wild-type Col-0, the cortical infection of *atg8e* and *urm1*, which showed more susceptible in soil culture infection, were not observed earlier than that of Col-0, but numerous secondary plasmodia could be observed at 3 dpi. While the cortical infection of the *tcp9* mutant was significantly earlier, few secondary plasmodia could be observed at 12 h post inoculation. On the other hand, the materials that showed less susceptibility in soil culture infection, such as *pme17* and *skp1*, which were not significantly different from Col-0. The observed infection time points of *pub15* were not significantly different, but the number of infected cortex cells was significantly less than that of Col-0 at the same points. In the case of *erf034*, the performance of hydroponic culture infection was also significantly different from that of Col-0. Not only was the time of cortical infection delayed, but also the state of secondary plasmodia in the cortical infection could be observed. In Col-0 and other susceptible materials, the secondary plasmodia of *P. brassicae* developed so fast that they took up most of the infected cells, or even filled a whole cortical cell, while in *erf034*, the secondary plasmodia were mostly in an undeveloped or early developing state just entering the root cortex cells, which was consistent with the results observed at 3 and 5 dpi.

#### 3.3.3 The *erf034* mutant has significant disease tolerance under high concentrations *P. brassicae* inoculation

**Figure 7:**
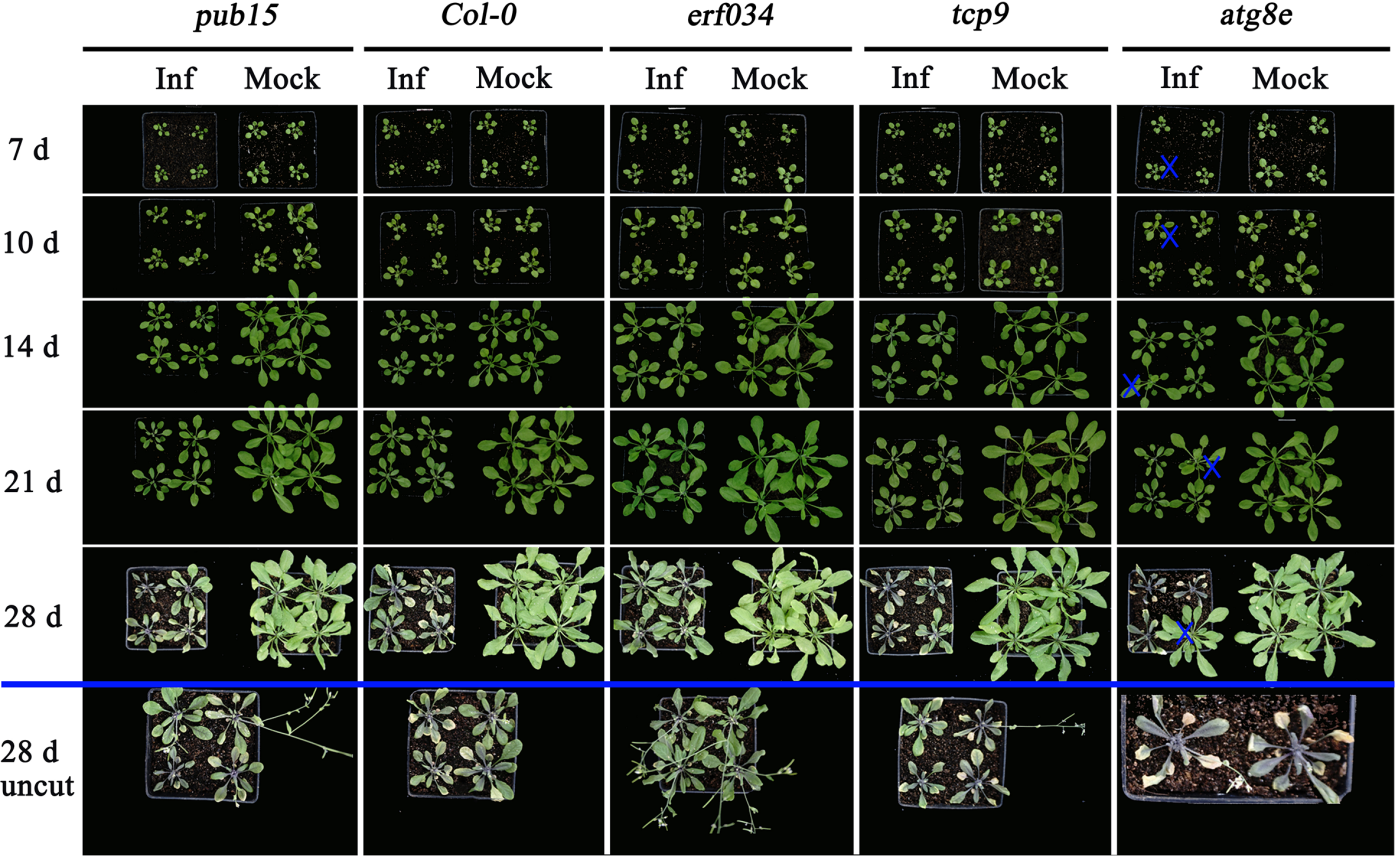
Clubroot disease development in *A. thaliana* for retesting. The plants below the blue line indicates the uncut above-ground portion, “×” indicates the single plants inoculated with sterile water in the *atg8e* treatment group.

Combining the results of soil culture and hydroponic culture infection, it can be tentatively concluded that *erf034* was an Arabidopsis mutant with the most prominent CR characteristic relative to wild-type Col-0 and other mutants, *pub15* was able to show some level of resistance, while *tcp9*, *urm1*, and *atg8e* may be more susceptible. To confirm this phenotype, we performed the resistance retesting with higher concentrations of resting spores and continuously recorded the phenotypes of each growth period. As shown in **Figure 7**, there were no significant phenotypic differences between the treatment (Inf) and control groups (Mock) for all materials at 10 dpi, while at 14 dpi, differences in plant size began to appear in two treatments of each material. The growth of infected plants was inhibited and their size was significantly smaller than that of control plants. This difference was further amplified by 21 dpi. Among the different materials, *tcp9* and *atg8e* showed stronger growth inhibition than *pub15* and Col-0, and this difference was more pronounced relative to *erf034*. These intergroup differences were also more pronounced at 28 dpi. For the sake of observation, the phenotypes at 28 dpi we listed were the photographic records of the treatment and control groups after stem cutting, while the uncut treatment groups showed that there were extremely obvious phenotypic differences in the two groups of different materials. On the one hand, *erf034* can shoot and flower normally and produce silique despite the stress of infection. On the other hand, the differentiation of flower buds was inhibited, and the number of shoots was less than that of the control group, especially the wild-type Col-0 was almost unable to shoot normally at 28 dpi. The infected plants were dwarf, the leaves started to wilt, and turned purple or yellow. The *pub15* varied considerably among individuals, with plants capable of normally flowering or dwarf and purple, which were similar to the phenotype of the more susceptible *tcp9*, but the overall growth and development were significantly inhibited. The susceptible plants *atg8e* were more extreme, showing purple leaves and even withering of the whole plants. In summary, the mutant *erf034* had the best CR phenotype in response to *P. brassicae* infection.

### 3.4 ERF034 is a transcriptional activator in response to biotic stresses

**Figure 8:**
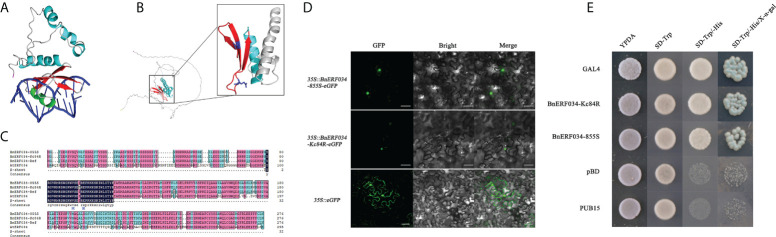
Bioinformatics analysis and related functional analysis of ERF034. **(A)** Conserved structural domain elements of ERF transcription factors. **(B)** Structure prediction of *At*ERF034 and characterization of its ERF structural domain. **(C)** Comparison of the ERF034 and β-sheet sequences from 855S, Kc84R and *A*. *thaliana*. **(D)** Subcellular localization of *Bn*ERF034-eGFP, scale bars represent 50 μm. **(E)** Transcriptional activation activity verification of *Bn*ERF034.

To further clarify the role of *ERF034* in the process of clubroot disease, relevant bioinformatics analysis and functional exploration of *ERF034* was performed. *ERF034* was abundantly expressed in the roots of *B. napus* and *A. thaliana* during the seedling stage (**Supplementary Figure 3**), and *AtERF034* showed down-regulated expression in various biotic stress states (**Supplementary Figure 4**). Transcription factors of the ERF family generally contain a highly conserved ERF domain, which is composed of 4 α-helices and 3 β-sheets, of which 1 α-helix and 3 β-sheets at the N-terminal are the key to the interaction of proteins with cis-elements (**Figure 8A**). The Valine 14 and Glutamicacid 19 on β-sheet are required for binding to cis-elements, and the flanking sequences affect the efficiency of ERF protein binding to cis-acting elements (Tournier et al., 2003; Canella et al., 2010). According to the structural prediction, *At*ERF034 had this typical ERF domain (**Figure 8B**). Furthermore, the protein sequences encoded by *BnERF034* did not differ much in the two materials, especially the β-fold sequence, encoded the ability to bind specific motifs was highly consistent (**Figure 8C**). This implies that the differences of resistance levels in different materials were not caused by functional differences of ERF034. To further clarify the location of *Bn*ERF034, *Bn*ERF034-855S-eGFP, and *Bn*ERF034-Kc84R-eGFP were expressed in *N*. *benthamiana* leaves, and fluorescent signals were detected by fluorescence microscope. The results showed that enhanced green fluorescent protein (eGFP) fluorescent signals were localized in the cell nucleus (**Figure 8D**). The transactivation abilities of *Bn*ERF034-855S and *Bn*ERF034-Kc84R were analyzed using a yeast assay system. As shown in **Figure 8E**, the transformants harboring GAL4, *Bn*ERF034-855S, and *Bn*ERF034-Kc84R grew well, while the cells containing pBD and PUB15 could not grow on the same medium. The X-α-gal staining for β-galactosidase activity showed blue in cells having GAL4, *Bn*ERF034-855S and *Bn*ERF034-Kc84R. These results indicate that the *Bn*ERF034-855S and *Bn*ERF034-Kc84R have transactivation capacity.

### 3.5 Heterogeneous expression of *BnERF034* enhanced the virulence of *P. brassicae*

**Figure 9:**
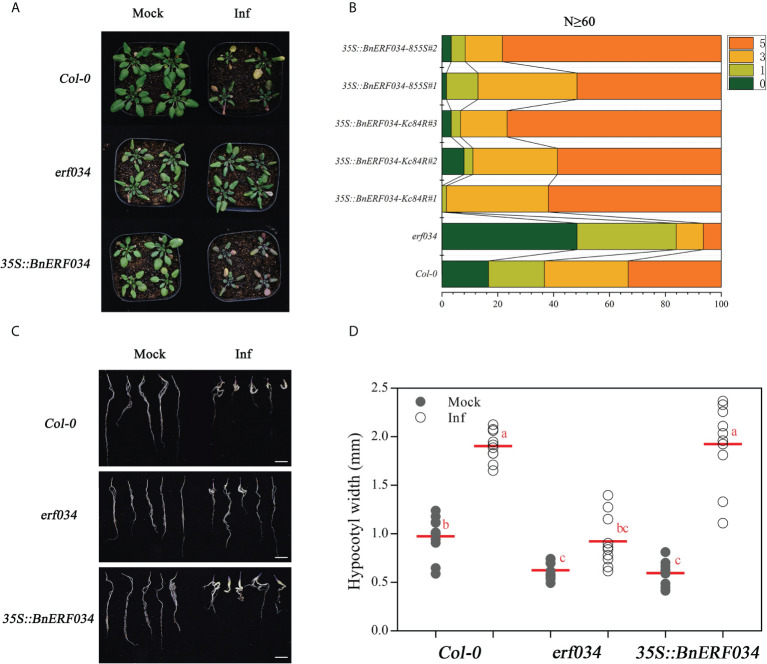
*BnERF034* enhanced the severity of clubroot disease in *A*. *thaliana*. **(A)** Above-ground phenotype of *A. thaliana* Col-0, *erf034*, and transgenic lines of *BnERF034* at 21 dpi. **(B)** Results of disease survey, 0-5 indicates disease grade. **(C)** Root phenotypes of *A*. *thaliana* materials at 28 dpi, scale bars represent 2 cm. **(D)** Hypocotyl width of different *A*. *thaliana* materials at 28 dpi in sterile water-inoculated (Mock) and *P. brassicae*-inoculated (Inf). Scatter plots present individual hypocotyl width measurements (10 replicates for each treatment), calculated means and SEs. Different letters indicate significant differences between means (Tamhane’s test, *p*< 0.05).

Additionally, we generated transgenic *A. thaliana* lines expressing *BnERF034* (from 855S and Kc84R, respectively) to further investigate the effect of *BnERF034* in clubroot disease. The positive plants and expression of *BnERF034* were confirmed by Basta solution, PCR and RT-qPCR, respectively (**Supplementary Figure 5**). All transgenic lines, mutants and wild type Col-0 were inoculated with *P. brassicae*. Above-ground partial phenotype photography and the occurrence of clubroot disease were investigated at 21 dpi. Root phenotypes were photographed and collected at 28 dpi. The results showed that the *BnERF034* transgenic plants were more severely susceptible to the clubroot disease under the same infection conditions (**Figure 9A**). In the wild type Col-0, the percentage of medium to severe galls (disease classes 3 and 5) was 63.3%, and the percentage of small galls (disease classes 0 and 1) was 36.7%. In the *BnERF034* heterogeneous expression lines, the percentage of medium to severe galls was higher, with a value of 98.4% in *35S::BnERF034-Kc84R*#1, 88.89% in *35S::BnERF034-Kc84R*#2, 93.33% in *35S::BnERF034-Kc84R*#3, 87.09% in *35S::BnERF034-855S*#1 and 91.66% in *35S::BnERF034-855S*#2. The percentage of medium to severe galls with a value of 16.13% in *erf034* mutants (**Figure 9B**). On the other hand, the hypocotyl width of infected plants was significantly decreased in the *erf034* mutant (on average by 62.25 at 28 dpi; **Figures 9C, D** **)**, and significantly increased in the *BnERF034* transgenic lines (on average by 262.07% at 28 dpi; **Figures 9C, D** **)**, in relation to Col-0 controls. These results illustrate that *BnERF034* enhanced the virulence of *P. brassicae* and promoted plant susceptibility to clubroot disease in *A. thaliana*, and further suggested that ERF034 was an important factor controlling CR trait and exhibited the characteristics of negative regulation.

**Figure 10:**
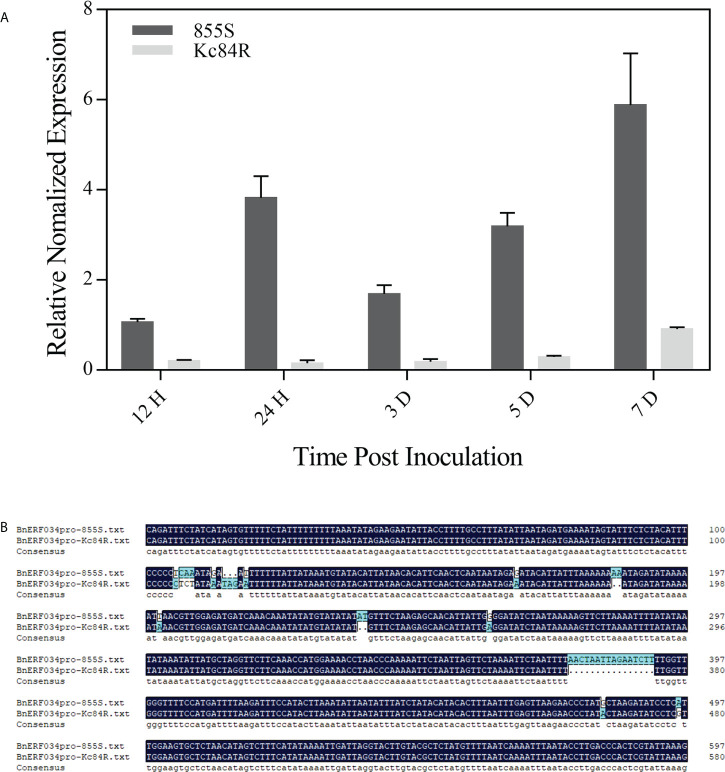
Expression patterns and predicted promoter regions of *BnERF034* in Kc84R and 855S. **(A)** Expression patterns of *BnERF034* in Kc84R and 855S in hydroponic culture infection with *P. brassicae*. **(B)** Sequence alignment of predicted promoter regions of *BnERF034* in Kc84R and 855S.

To explore the relationship between the CR trait and *ERF034*, we further compared the transcript level of *ERF034* in response to the *P. brassicae* infection between Kc84R and 855S in hydroponic culture. The results showed that the transcript level of *BnERF034* in 855S gradually increased over time, but it did not strictly increase with the infection stage. There was no significant change in all stages in Kc84R, and compared with 855S, the transcript level of *BnERF034* was significantly lower (**Figure 10A**). Combined with the data from soil culture (**Figure 4B**), the transcript levels of *BnERF034* in Kc84R at each time point we tested were the same significantly lower than 855S. We further analyzed the segments containing the promoter sequence in Kc84R and 855S according to the BSA-Seq files and found that Kc84R had many deletions and mutations in the segment predicted to be *BnERF034* promoter element, relative to 855S (**Figure 10B**). In particular, in the predicted promoter region from 1,779 bp to 1,729 bp upstream of the 5’-UTR, there was an AT deletion and a G→A base substitution, and there was also a 16 bp fragment deletion downstream adjacent to this region, which may directly affect the transcript level of *BnERF034*.

## 4 Discussion

With the development of modern agriculture, *P. brassicae* has rapidly spread among major oilseed producing areas in China. Clubroot disease has become one of the most serious diseases affecting *Brassicaceae* crop production (Dixon, 2009; Chai et al., 2014). Therefore, it is urgent to choose more economical and effectively management measures to control clubroot disease. Clubroot disease resistance breeding has proven to be a powerful approach, however, there are still two important problems for the resistance breeding: one is the lack of CR resources, especially in *B. napus* (Werner et al., 2008); Another is the complex genetic basis and relatively genetic variation of *P. brassicae*. These make it necessary to screen and breed corresponding CR materials for different *P. brassicae* isolates (Strelkov et al., 2016). The pathotype in Guanghan, Sichuan province (*Pb*Gh) used in this study has been identified as Pb3, according to the sinitic clubroot differential set (SCD) system (Pang et al., 2020). In our study, Kc84R is a selected line of *B. napus* “Kc84-1,” which is obtained from ARTP mutagenesis and has stable *Pb*Gh CR trait. In view of the current status of CR genes in *B. napus*, the CR locus in Kc84R has great potential to be studied. In this study, the CR locus mapping population was performed with BSA-seq approach and SNP genotyping. Before that, the parents (“Kc84R” and “855S” respectively), the F_1_ and F_2_ populations were inoculated with the *Pb*Gh isolate, respectively. The phenotype of F_1_ and F_2_ populations infected showed that the CR trait was controlled by a dominant locus (**Table 2**, **Figure 2A**). A single peak was identified on the A03 chromosome, and the CR locus was further narrowed down to an interval of 267 kb. This result differed from previous reports of resistance to clubroot disease in *B. napus*.

Generally, plant disease resistance refers to the interaction between plant innate immune system and pathogens, that is, the interaction of pathogen-associated molecular patterns (PAMPs) and effector proteins with PTI (PAMP-triggered immunity) or ETI (effector-triggered immunity) (Deng et al., 2020). Therefore, the current mapping of disease resistance genes generally refers to identifying genes that can participate in the plant immune system, and then exploring their role in the disease resistance process. Firstly, there are numerous immune receptors consisting of receptor-like proteins (RLPs) and receptor kinases (RKs) on the cell surface of host plants, which can recognize the PAMPs of invading pathogens (Tang et al., 2017; Zhou and Zhang, 2020). A series of immune responses are subsequently activated, such as calcium influx, reactive oxygen species (ROS) burst, production of defense hormones and activation of mitogen-activated protein kinase (MAPK) cascades, etc. (Tang et al., 2017). In addition, plant intracellular immune receptors can be classified into three major subgroups according to their N-terminal domains: TIR-NBS-LRR (TNL) proteins carrying a Toll Interleukin-1 Receptor (TIR) domain, CC-NBS-LRR (CNL) proteins carrying a coiled coil (CC) domain, and RPW8-NB-ARC-LRR (RNL) proteins carrying a RPW8-like (CC R) domain (Wang and Chai, 2020), all of which can specifically sense various pathogen effector proteins that are delivered into the plant cell (Zhou and Zhang, 2020). Subsequently, the corresponding downstream defense responses are also activated, such as programmed cell death or called hypersensitive response (HR), SA biosynthesis, ROS burst, MAPK cascades and other immune responses (Zhang et al., 2017; Yuan et al., 2021). It turns out that in both immune pathways, plants ultimately rely on various immune responses to resist the infection by pathogens. The mapping of disease resistance genes is the search for genes involved in plant immune responses. Although the reported CR loci either directly encode (only two CR genes, namely, *CRa* and *Crr1a*) or contain TIR-NB-LRR proteins, the mechanisms of resistance involved in these disease resistance proteins have rarely been studied.

As there are no common CR genes belonging to TNL gene families in the target region, we need to perform functional annotation and screening of all candidate genes. It is well known that the interaction between host plants and pathogens is a complex process. In face of pathogens invasion, host plants have evolved various ways to resist the attack, including but not limited to autophagy (blocking further colonization by discarding the infected part) (Zhang et al., 2021), ubiquitin pathway (targeting certain proteins are involved in disease resistance response, enhancing the system’s disease resistance response) (Christians et al., 2009; Li et al., 2012), and some broad-spectrum resistance, such as systemic acquired resistance (SAR), in which an important defense hormone, salicylic acid (SA), plays a major role (Century et al., 1997; Zheng et al., 2015). By analyzing the candidate genes, we found that *ATG8E* annotated to participate in autophagy, *PUB15*, *URM1* and *SKP1* involved in ubiquitination, and the transcription factor *TCP9* is related to salicylic acid synthesis (Wang et al., 2015). However, these *A. thaliana* lines corresponding to the candidate genes showed relatively less effect on CR. For example, the 742nd position of the *PUB15* encoding sequence in Kc84R, mutated from C to T, has been early terminated (**Supplementary Figures 6A, B** **)**. The functional domain of *BnPUB15* is mainly composed of 3 ARM domains, which contains many polypeptides binding sites (**Supplementary Figure 6C**). This implies that *BnPUB15* may regulate the clubroot disease by targeting a certain protein. We carried out the infection tests with the relevant lines of *BnPUB15*, and the results showed that the transgenic lines were not more obvious than the wild-type Col-0, some of them showed even more susceptible (**Supplementary Figure 6D**). *BnTCP9* was the largest difference in transcript level between Kc84R and 855S (**Figure 4B**). In the previous infection experiments, we also found that *tcp9* mutants showed earlier infected symptoms (**Figures 5A**, **6**). Correlation analysis and functional verification experiments showed that *BnTCP9* was a nuclear localized protein, its homologous sequence in *A. thaliana* had a typical transcription factor domain (**Supplementary Figures 7A, B** **)**. Additionally, TCP9 can bind to the promoter region of *ICS1* and positively regulate the expression of *ICS1* (Wang et al., 2015) (**Supplementary Figure 7E**). But similar to the results of other studies, the SA pathway cannot resist the infection of *P. brassicae* alone, and a relatively high content of SA can only relatively alleviate the disease symptoms (Lovelock et al., 2016). Therefore, even though the infected *BnTCP9* transgenic lines had delayed leaf senescence compared with Col-0 and *tcp9*, the galls were not significantly reduced compared to Col-0 (**Supplementary Figures 7C, D** **)**. Furthermore, we also constructed transgenic lines for several other genes, but some were unsuccessfully transformed in the construction of transgenic lines and others were missing during the passaging process due to various problems. The mining of CR-related genes in target region still needs to be further developed.

In our study, ERF034 is a negative regulator participating in clubroot disease, but the corresponding regulation pathway is still unclear. ERF is a major sub-family of AP2/ERF transcription factor, and generally contains only an AP2/EREBP domain. The cis-acting elements that can be combined mainly include two categories: GCC boxes and DRE/CRT (DRE: Dehydration-Responsive Element, CRT: C Repeat); (Qin et al., 2004). GCC box (conservative sequence is GCCGCC) exists in a large number of *PR* gene promoters (Büttner and Singh, 1997). DRE/CRT components (conservative sequences are CCGAC) mostly exist in the genes involved in low temperature response or are drought induced (Liu et al., 1998). ERF transcription factors can regulate the expression of most target genes through these two types of cis-acting elements with occasional exceptions (Chakravarthy et al., 2003). Additionally, ERF transcription factors play an important role in both abiotic stress responses and biotic stress responses in plants. Previous studies have used knockout mutants and overexpression materials of the ERF family genes to verify their function in the disease resistance response of *A. thaliana*. For example, the resistance of plants overexpressing *AtERF4* to *Fusarium oxysporum* was significantly reduced, while the resistance of loss-of-function mutant *erf4* was enhanced, indicating that *AtERF4* can negatively regulate Fusarium wilt resistance, and overexpression of *AtERF2* can improve *A. thaliana* resistance to Fusarium Wilt (McGrath et al., 2005). Onate-Sanchez et al. tested *erf14* mutants and *AtERF14* overexpressing lines and found that loss of *ERF14* function resulted in reduced resistance to Fusarium wilt in *A. thaliana*, whereas overexpression enhanced this resistance (Oñate-Sánchez et al., 2007). In addition, the overexpression of *AtERF14* can suppress JA-ethylene responsive *AtPDF1.2* while activate SA-responsive *AtPR1/5* genes (Zhang et al., 2016). *AtERF019* can interact with the transcriptional co-repressor NINJA, and transgenic Arabidopsis overexpressing *ERF019* is more susceptible to *Botrytis cinerea* (Huang et al., 2019). All these studies have shown that ERF family transcription factor is highly related to biotic stress. As for ERF034, *At*ERF034 is a nuclear-localized transcription factor, which can bind the core cis-acting element DRECRTCOREAT, and upregulate the promoter activity of *CESA1* (cellulose synthase catalytic subunit 1) (Schwacke et al., 2007; Saelim et al., 2019). *At*ERF034 can also interact with *TMO6* (AT5G60200) and participate in the regulatory network of Arabidopsis root development (Brady et al., 2011). Furthermore, the regulatory network analysis of *BnERF034* homologous participating in *A. thaliana* and *B. rapa* showed that the genes annotated as *RAP2.7* (*TOE1*) and *TOE2* were present in both materials (**Supplementary Figure 8**, **Supplementary Tables 9**, **10**). The miR172/TOE1-TOE2 is an important regulatory module in plants, involved in plant development regulation, morphogenesis and innate immunity (Zou et al., 2018; Werner et al., 2021). There has been reported that this module in response to *P. brassicae* infection in *B. napus* (Li et al., 2021a). In summary, the exploration of the CR mechanism involving *ERF034* can be carried out from these three aspects: (i) hormone regulation associated with *ERF034*, including ethylene, SA, jasmonic acid (JA), etc.; (ii) target genes that *ERF034* can regulate through cis-acting elements; (iii) modules related to plant immunity involving *ERF034*, such as miRNAs, etc. Therefore, in-depth exploration of *ERF034* is helpful to explain its involvement in the CR.

## Data availability statement

The data presented in the study are deposited in the Genome Sequence Archive in National Genomics Data Center repository, accession number CRA008099.

## Author contributions

MW and XJ designed and directed the experiments. XJ and YS performed most of the experiments and analysed the data. XJ wrote the manuscript. All authors contributed to the article and approved the submitted version.

## Funding

This research was funded by The National Key Research and Development Plan, grant numbers 2016YFD0100202 and 2018YFD0100501, and Sichuan Province Breeding Project, grant number 2016NYZ0031.

## Conflict of interest

The authors declare that the research was conducted in the absence of any commercial or financial relationships that could be construed as a potential conflict of interest.

## Publisher’s note

All claims expressed in this article are solely those of the authors and do not necessarily represent those of their affiliated organizations, or those of the publisher, the editors and the reviewers. Any product that may be evaluated in this article, or claim that may be made by its manufacturer, is not guaranteed or endorsed by the publisher.
