# Supplementary material for: Mapping of a novel clubroot disease resistance locus in Brassica napus and related functional identification
Source: Front Plant Sci. 2022 Sep 28;13:1014376. doi: 10.3389/fpls.2022.1014376 (PMC9554558; doi:10.3389/fpls.2022.1014376)
Supplement: Supplementary file 1 [file DataSheet_1.docx]

Supplementary Material

# Supplementary Figures

**Supplementary Figure 1.** Methodology for grading clubroot disease investigations of *B. napus*.

**Supplementary Figure 2.** Methodology for grading clubroot disease investigations of *A. thaliana*.

**Supplementary Figure 3.** Expression levels of *AtERF034* and *BnERF034* in tissues and organs.

**Supplementary Figure 4.** Expression pattern of *AtERF034* under different pathogen stresses and expression levels of root response elicitations in *A. thaliana*.

**Supplementary Figure 5.** Screening and identification of transgenic lines of *BnERF034*.

**Supplementary Figure 6.** The related functional identification about *BnPUB15*.

**Supplementary Figure 7.** The related functional identification about *TCP9*.

**Supplementary Figure 8.** Predicted interacting proteins of ERF034.


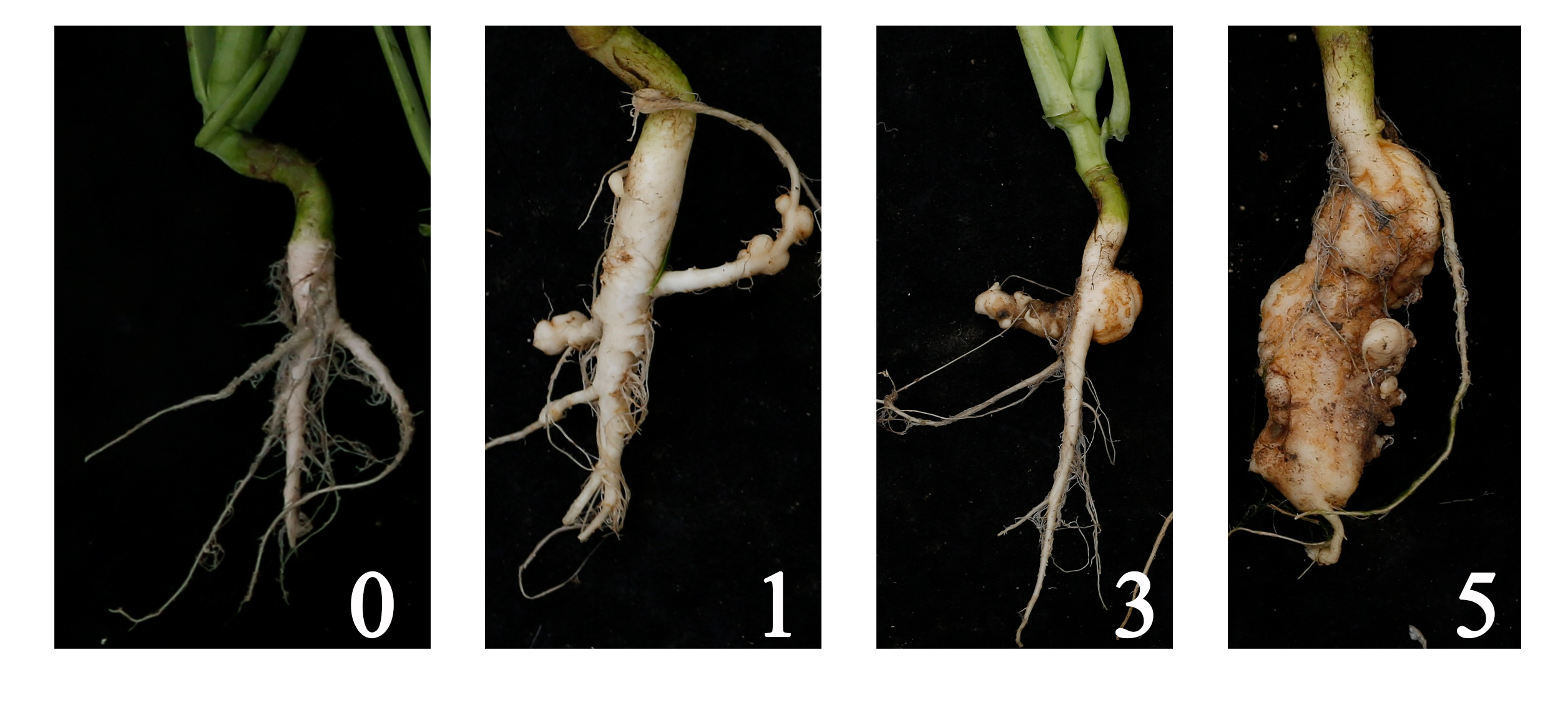
**Supplementary Figure 1.** Methodology for grading clubroot disease investigations of *B. napus*. “0,” “1,” “3,” “5” mean disease symptoms scores.


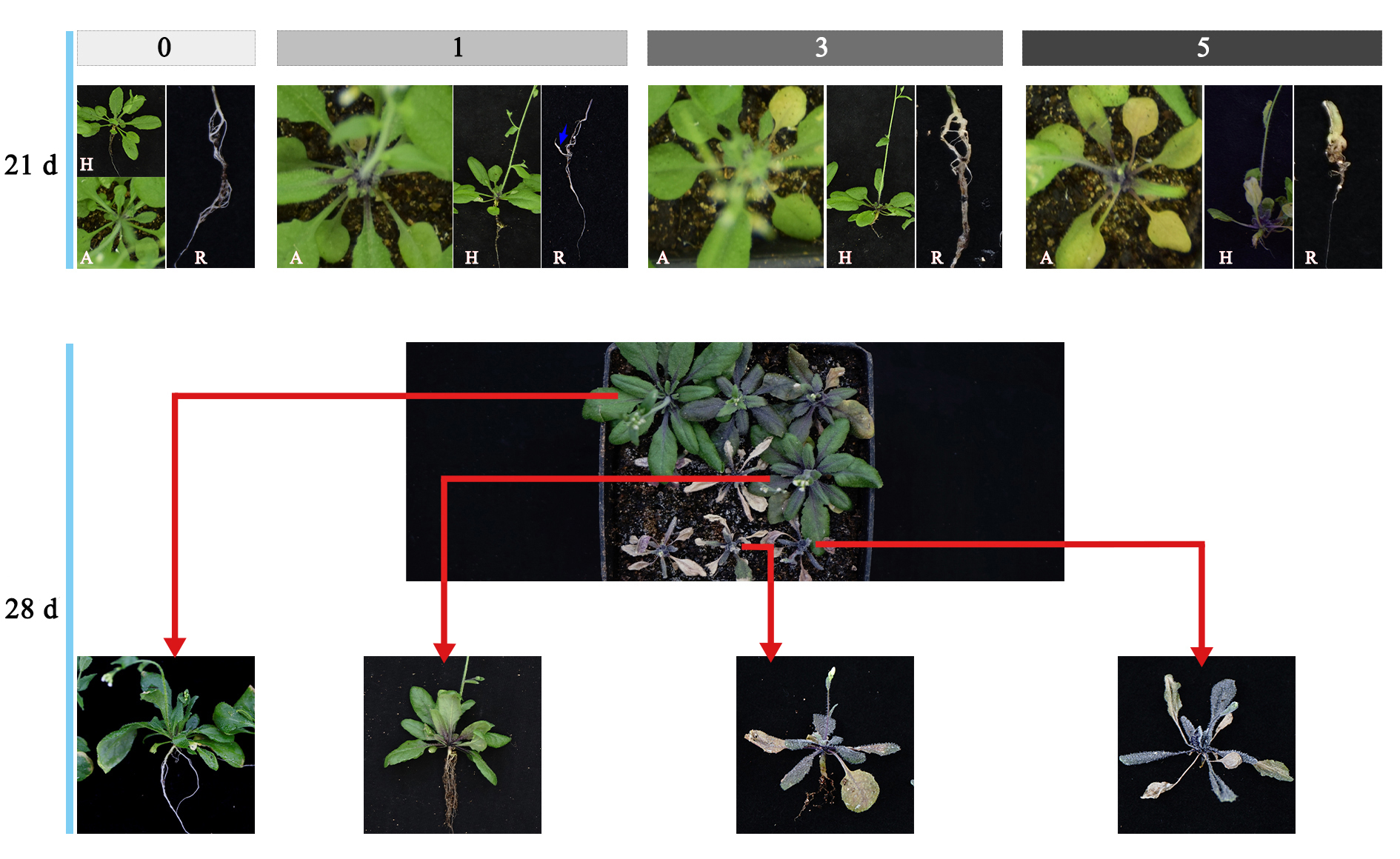
**Supplementary Figure 2.** Methodology for grading clubroot disease investigations of *A. thaliana*. H indicates the phenotype of whole plants; R indicates the roots; A indicates the above-ground section of plants; blue arrow indicates the root gall of grade 1. “0,” “1,” “3,” “5” mean disease symptoms scales.

**
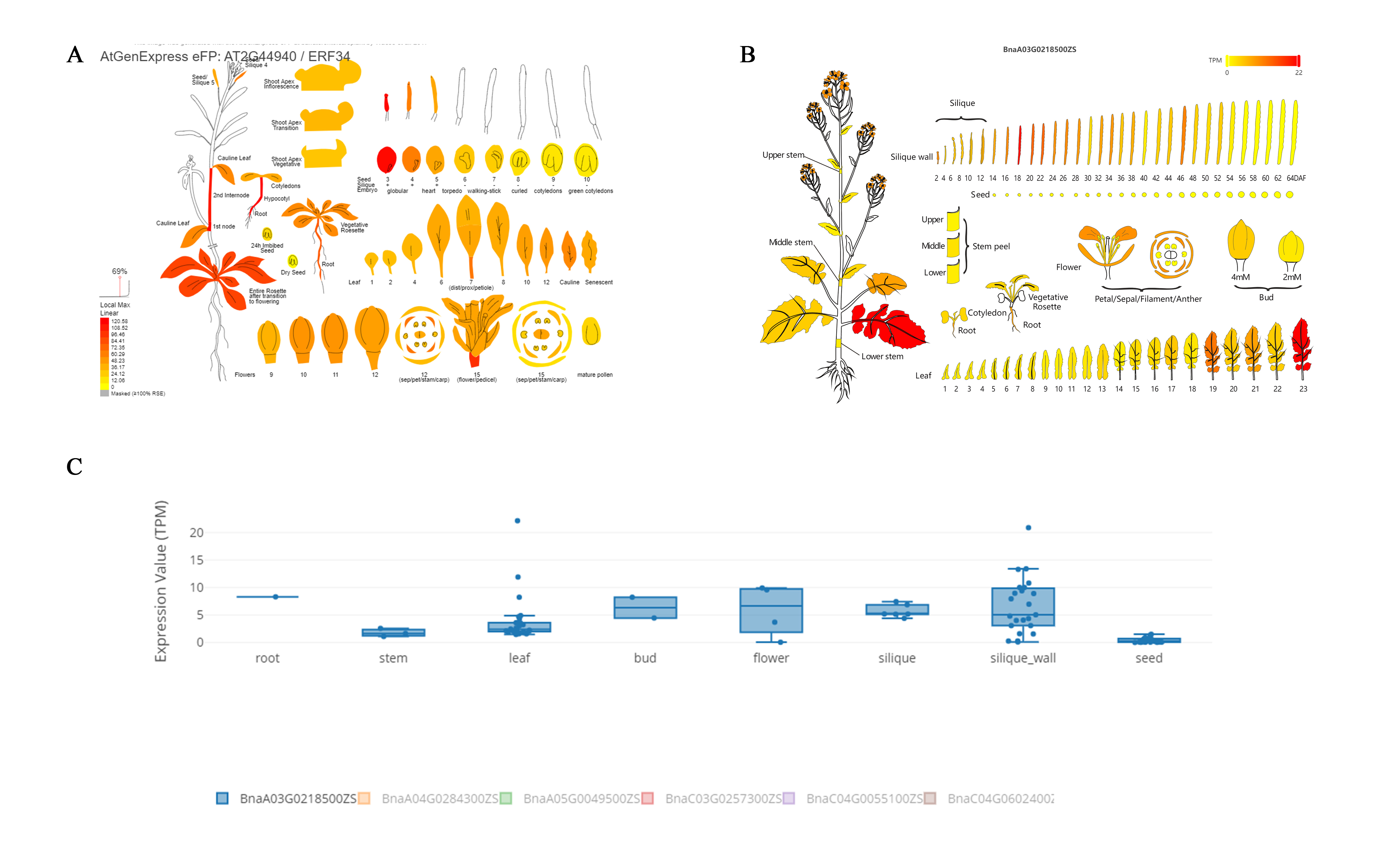
**

**Supplementary Figure 3.** Expression levels of *AtERF034* and *BnERF034* in tissues and organs. (A) Expression levels of *AtERF034* in *A. thaliana*; (B) Expression levels of *BnERF034* in *B. nupus*; (C) Expression levels of *BnERF034* in different organs.

**
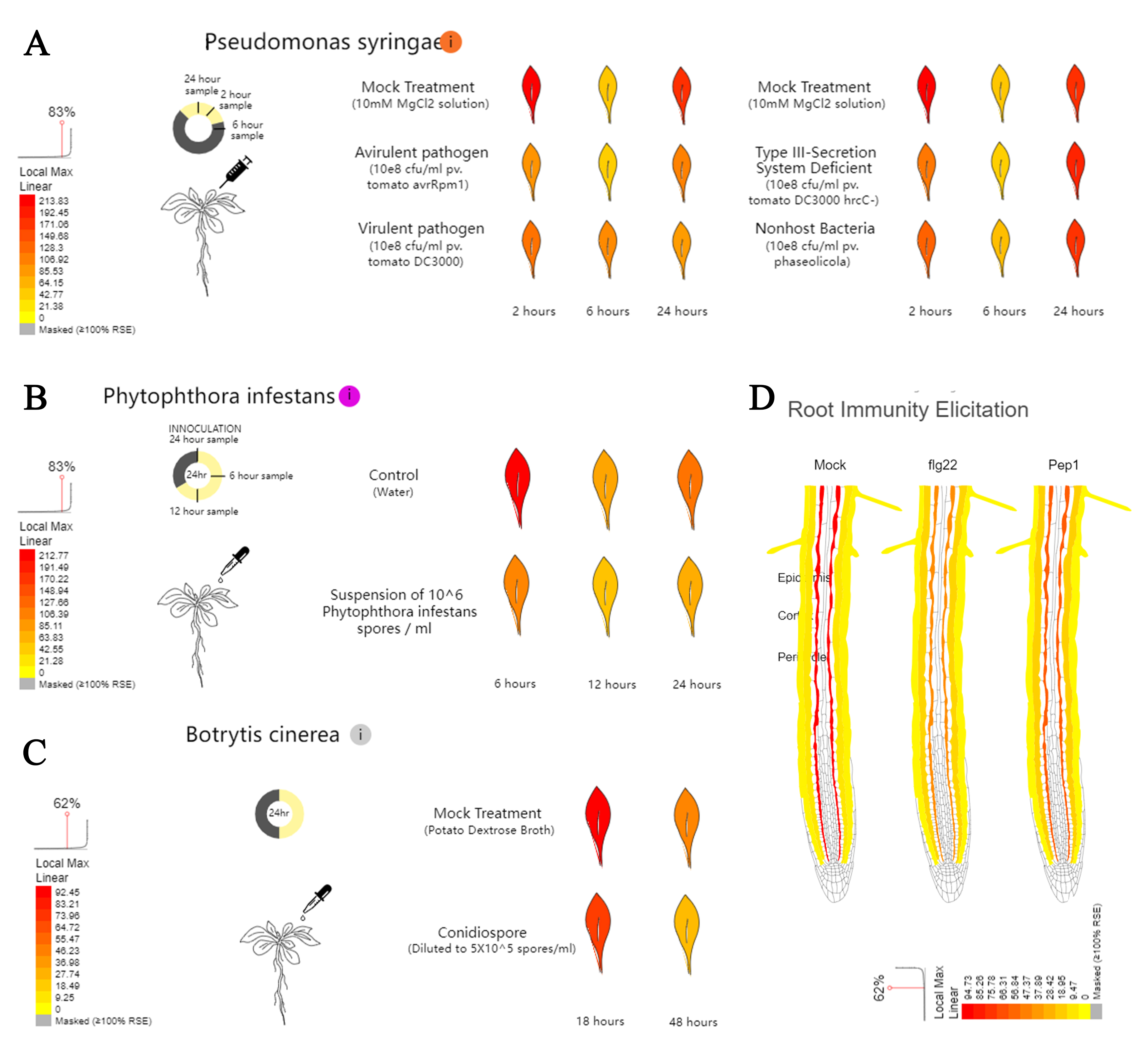
**

**Supplementary Figure 4.** Expression pattern of *AtERF034* under different pathogen stresses and expression levels of root response elicitations in *A. thaliana*. (A) treat with *P. syringae*; (B) treat with *P. infestans*; (C) treat with *B. cinerea*; (D) *A. thaliana* roots were treated with flg22 and Pep1.

**
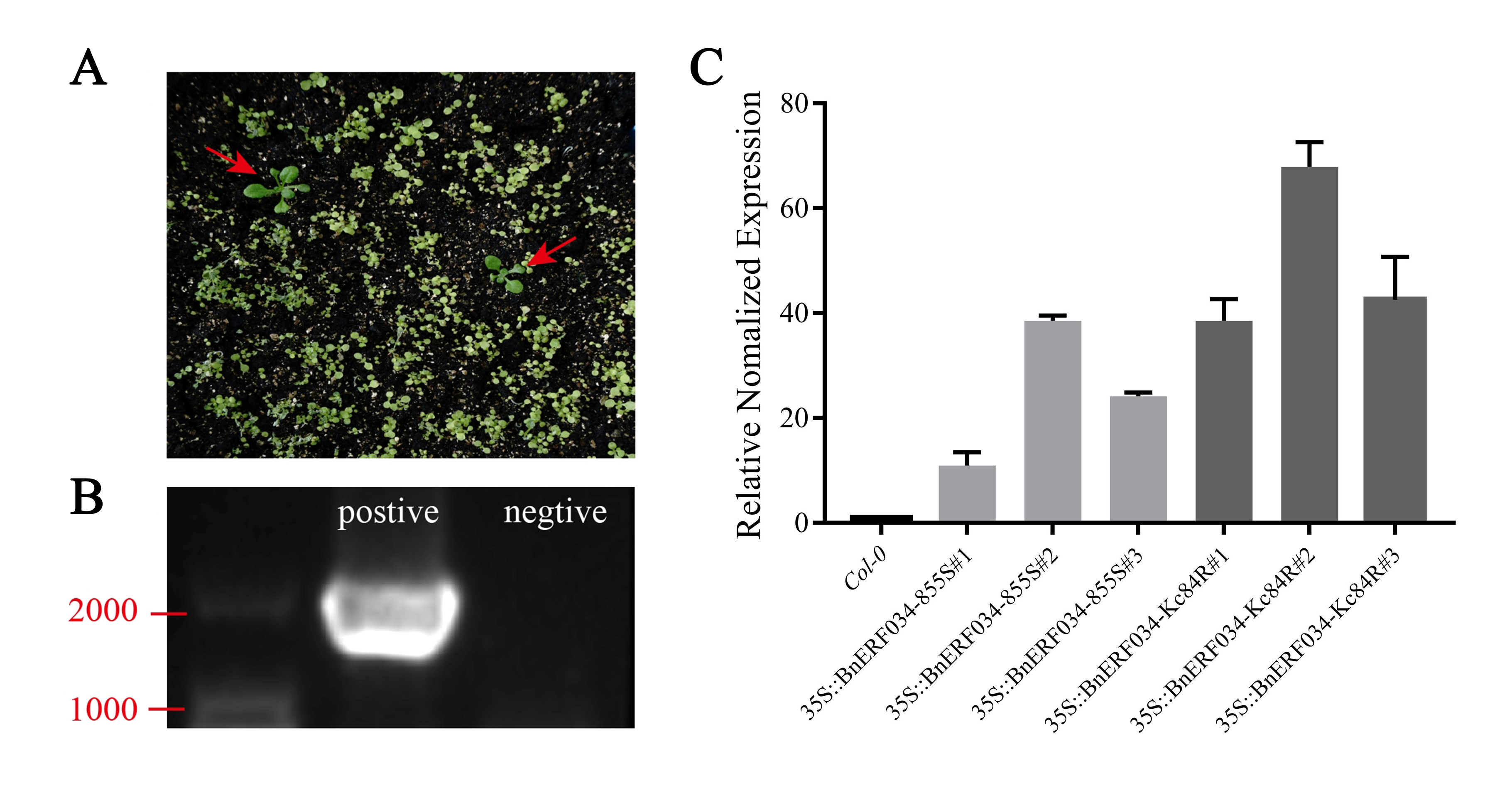
**

**Supplementary Figure 5.** Screening and identification of transgenic lines of *BnERF034*. (A) Basta screening of T_0_ generation seeds of transgenic plants in soil culture; (B) PCR identification of positive plants; (C) Transcript level identification of T_3_ generation positive plants.

**
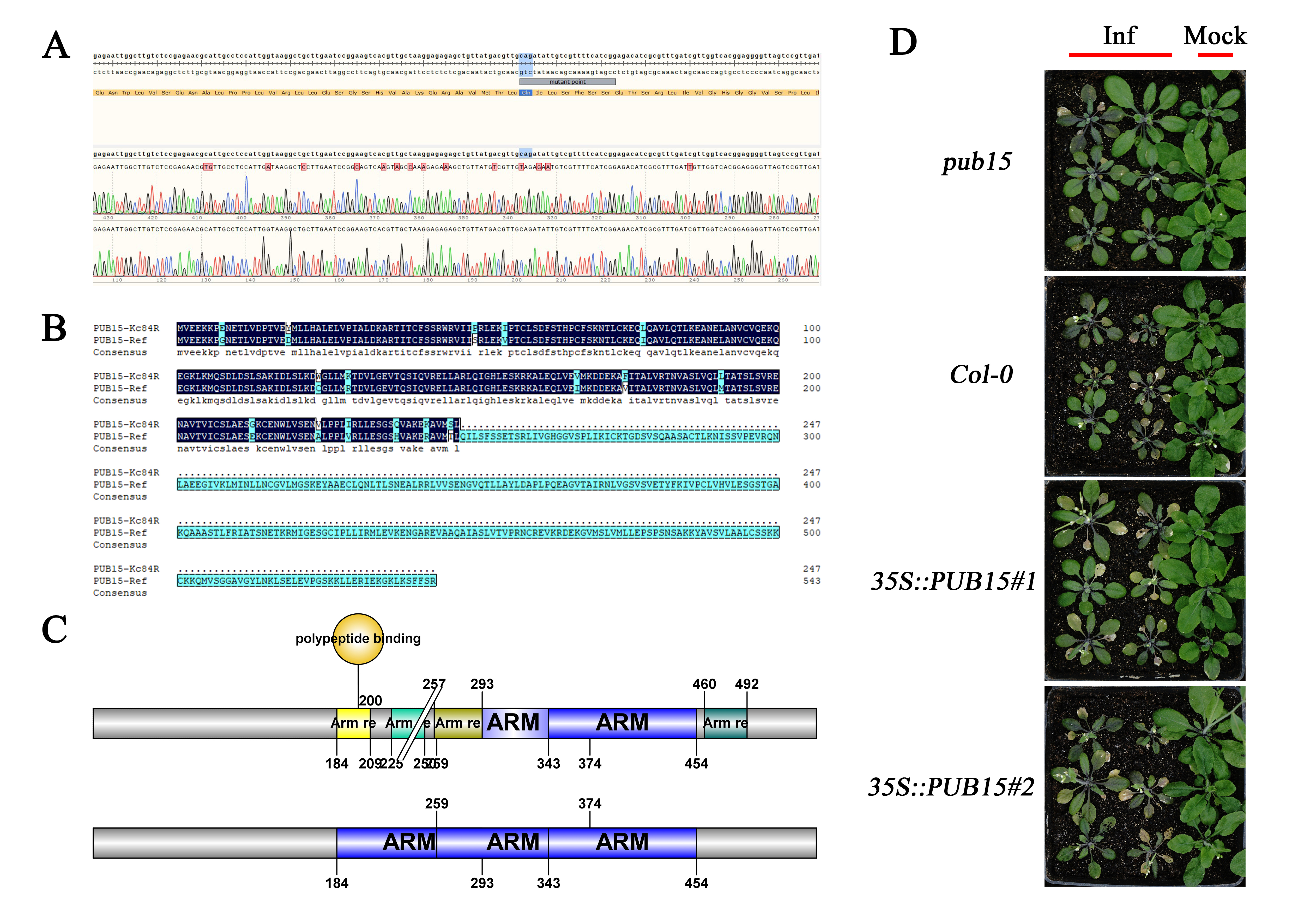
**

**Supplementary Figure 6.** The related functional identification about *BnPUB15*. (A) Partial sequence of *BnPUB15*: sequence comparison including stop loss in 855S and Kc84R. (B) Protein sequence comparison of *Bn*PUB15 and 855S (the same as Zhongshuang11); (C) Schematic diagram of the protein structural domain of *Bn*PUB15; (D) Above-ground partial phenotype of *A. thaliana* *Col-0*, *pub15*, *BnPUB15* transgenic lines 21 dpi with *P. brassicae*.


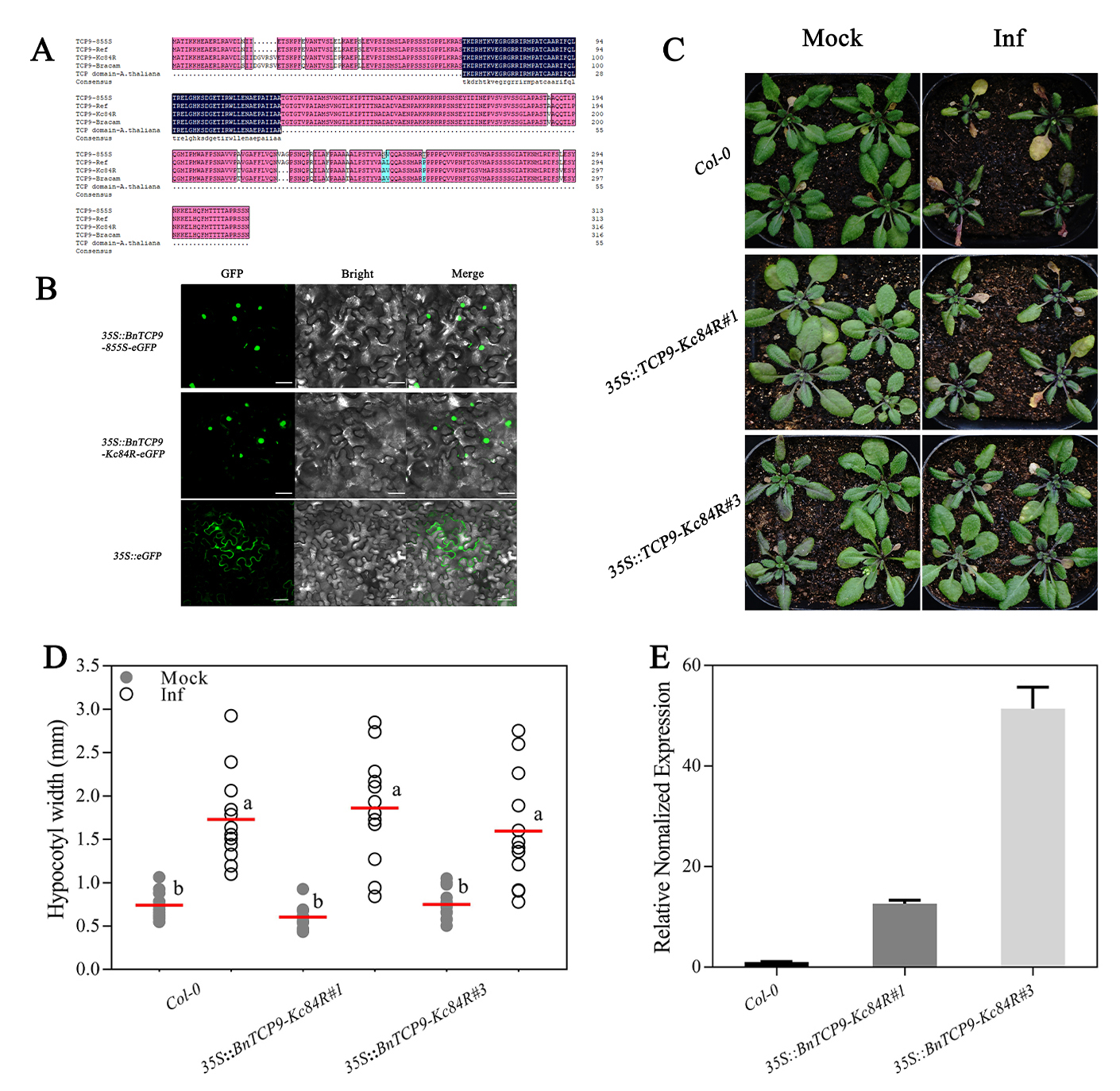


**Supplementary Figure 7.** The related functional identification about *TCP9*. (A) The protein sequences of TCP9 in 855S, Zhongshuang 11, Kc84R, *B. campestris* L., and the sequence alignment with TCP domain in *At*TCP9. (B) The results of subcellular localization of *Bn*TCP9-855S-eGFP and *Bn*TCP9-Kc84R-eGFP, pBI121-eGFP as control, scale bars represent 50 μm. (C) The *BnTCP9-Kc84R* transgene lines’ phenotype of above-ground parts after 21 dpi with *P. brassicae*. (D) Hypocotyl width of the Col-0 and *BnTCP9-Kc84R* transgenic lines at 28 dpi in sterile water-inoculated (Mock) and *P. brassicae*-inoculated (Inf). Scatter plots present individual hypocotyl width measurements (10 replicates for each treatment), calculated means and SEs. Different letters indicate significant differences between means (Tamhane’s test, *p*< 0.05). (E) Transcript levels of *AtICS1* in the Col-0 and *BnTCP9-Kc84R* transgenic lines at 21 dpi.

**
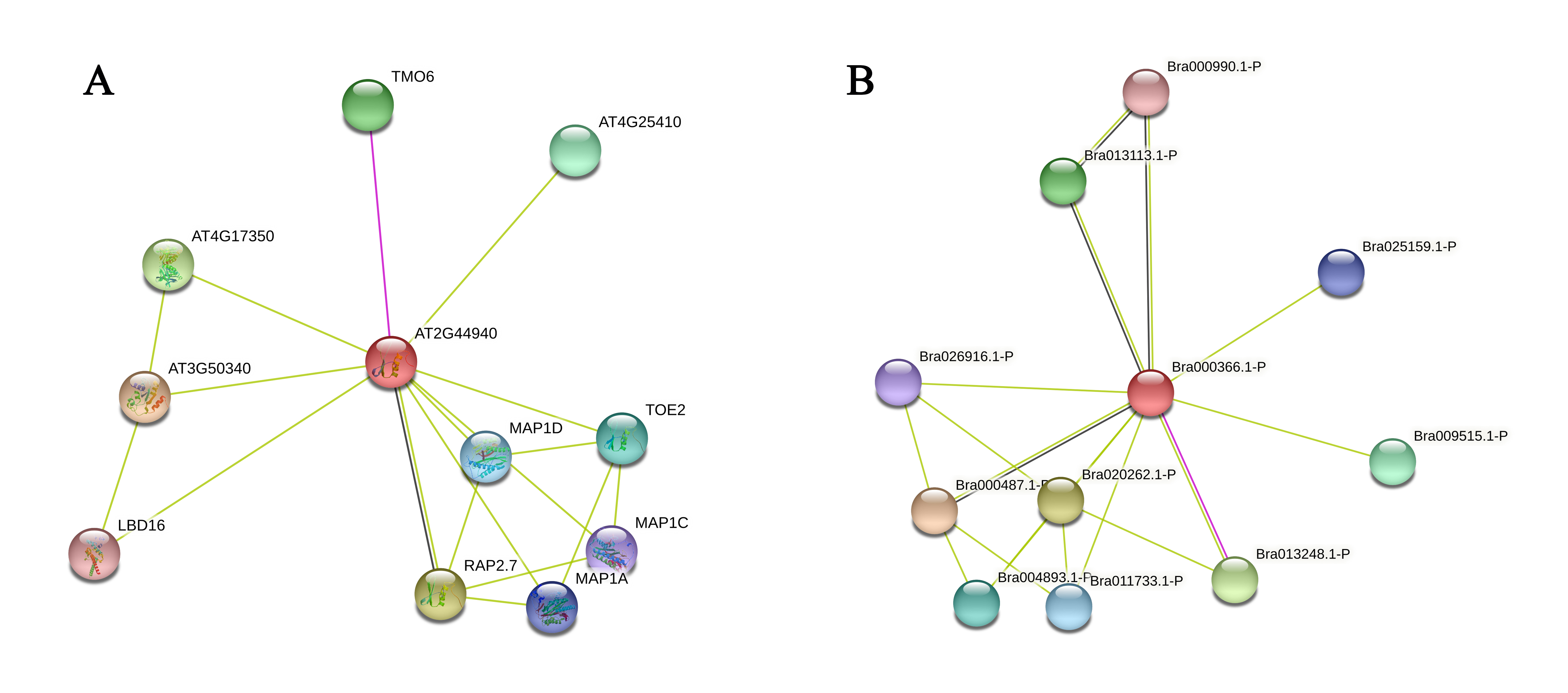
**

**Supplementary Figure 8.** Predicted interacting proteins of ERF034. (A) The predicted interacting proteins of *At*ERF034; (B) The predicted interacting proteins of *Br*ERF034; Red lines indicate interactions with experimental evidence, black lines indicate predictions of co-expression, green lines indicate predictions of interactions.
